# Combining unequal variance signal detection theory with the health belief model to optimize shared decision making in tinnitus patients: part 1—model development

**DOI:** 10.3389/fnins.2024.1451741

**Published:** 2024-12-04

**Authors:** Alexander E. Hoetink, Sarah Kaldenbach, Arnold Lieftink, Huib Versnel, Robert J. Stokroos

**Affiliations:** ^1^Department of Otorhinolaryngology and Head & Neck Surgery, University Medical Center Utrecht, Utrecht, Netherlands; ^2^UMC Utrecht Brain Center, Utrecht, Netherlands; ^3^Dutch Foundation for the Deaf and Hard of Hearing Child (NSDSK), Amsterdam, Netherlands

**Keywords:** tinnitus, patient related outcome measures, shared decision making, sound therapy, cognitive behavioral therapy, hearing loss, signal detection theory, health belief model

## Abstract

**Introduction:**

The results from different Cochrane studies justify considerable professional equipoise concerning different treatment options for tinnitus. In case of professional equipoise, Shared Decision Making (SDM) is an indispensable tool for guiding patients to the intervention that best fits their needs. To improve SDM we developed a method to assess the accuracy and utility of decisions made by tinnitus patients when freely choosing between different treatment options during their patient journey. The different treatment options were audiological care and psychosocial counseling.

**Methods:**

We developed a statistical model by combining Signal Detection Theory (SDT) with the Health Belief Model (HBM). HBM states that perceived severity of an illness is strongly related to sick-role behavior. As proxies for perceived severity, we selected hearing loss and Tinnitus Handicap Inventory (THI) score at baseline. Next, we used these proxies as predictors in linear regression models based on SDT to determine the likelihood ratio of true positive decisions (choosing a treatment option and experiencing an improvement of more than 7 points in THI-score) and false positive decisions (choosing a treatment option and experiencing an improvement of less than 7 points in THI-score) for audiological care and psychosocial counseling, respectively. Data was gathered in a prospective cohort of 145 adults referred for tinnitus care to an outpatient audiology clinic in the Netherlands. The participants were asked to decide freely on uptake of audiological care (provision of hearing aids with or without a sound generator) and uptake of psychosocial counseling. Logistic regression with Bayesian inference was used to determine the cumulative distribution functions and the probability density functions of true positive decisions and false positive decisions as function of hearing loss and baseline THI-score for both treatment options, respectively. With the cumulative distribution functions, we determined the accuracy of the decisions. With the probability density functions we calculated the likelihood ratios of true positive decisions versus false positive decisions as function of hearing loss and baseline THI-score. These likelihood ratio functions allow assessment of the utility of the decisions by relating a decision criterion to perceived benefits and costs.

**Results:**

Baseline THI-score drives decisions about psychosocial counseling and hearing loss drives decisions about audiological care. Decisions about psychosocial counseling are more accurate than decisions about audiological care. Both decisions have a low accuracy (0.255 for audiological care and  − 0.429 for psychosocial counseling), however. For decisions about audiological care the unbiased decision criterion is 37 dB(HL), meaning that a lenient decision criterion (likelihood ratio < 1) is adopted by patients with a hearing loss below 37 dB and a strict criterion (likelihood ratio > 1) by patients with a hearing loss exceeding 37 dB. For psychosocial counseling uptake the decision criterion is always strict, regardless of baseline THI-score. The distributions of the populations, that do and do not experience a clinically important change in THI-score, have unequal variances for psychosocial counseling, while they have almost equal variances for audiological care.

**Discussion:**

Combining SDT and HBM can help assess accuracy and utility of patient decisions and thus may provide valuable information that can help to improve SDM by combining patient related outcome measures, decision drivers, and perceived benefits and costs of a treatment.

## 1 Introduction

Subjective tinnitus is defined as the sensation of sound in the absence of an external sound source (Baguley et al., 2013). Symptoms range from relatively harmless annoyance, irritability and slight hearing difficulties to severe frustration, anxiety and even depression. The prevalence of chronic tinnitus in adults ranges from 4.7 to 19.3% (Jarach et al., 2022). The etiology of tinnitus can vary from relatively easily medically treatable problems such as ear wax or conductive hearing loss, to untreatable or even clinically undetectable cochlear or neuro-otological damage due to, e.g., noise trauma, presbycusis, or ototoxicity. The underlying brain mechanisms that cause the development of subjective and chronic tinnitus are discussed in several reviews and studies, for example (De Ridder et al., 2014; Galazyuk et al., 2012; Knipper et al., 2020; Langguth et al., 2013). Different brain regions are involved in tinnitus that include both auditory and non-auditory brain and brain stem structures (De Ridder et al., 2014; Langguth et al., 2013): (1) the salience network that comprises the anterior insula, anterior cingulate, and thalamus needs to be coactivated, by which frontal cortical networks will be directed to produce a conscious auditory perception; (2) a non-specific distress network that comprises the anterior cingulate cortex, the anterior insula and the amygdala has been shown to be activated differently in participants with tinnitus experiencing high versus low distress; (3) memory mechanisms may play a role in the persistence of the phantom percept, and in the reinforcement of the associated distress. A new framework has been proposed by Krauss et al. (2016), Schilling et al. (2023), and Sedley et al. (2016), that explains perpetuation of a tinnitus percept based on predictive coding of auditory input by the brain in combination with focused attention as top-down mechanisms and stochastic resonance and central gain increase as bottom-up mechanisms.

Broad consensus exists that chronic subjective tinnitus management requires a multidisciplinary approach with attention to medical, audiological, psychological and social–emotional issues (Cima et al., 2014). The initial focus is to rule out medically treatable and/or serious causes. In addition to this medical focus, it is recognized that it is important to provide sympathetic, compassionate care from the start instead of demonstrating an attitude that “nothing can be done about tinnitus and you have to learn to live with it” (Marks et al., 2019). As a cure for tinnitus is not available at this moment, other options remain that focus on symptom reduction (Rademaker et al., 2021a). Recommended therapy options are based on psychological intervention (e.g., Cognitive Behavioral Therapy [CBT] or Mindfulness), audiological intervention (e.g., sound enrichment devices or hearing aids), or a combination (Cima et al., 2019).

A historical overview of the development of psychological interventions is provided in Cima et al. (2014). These interventions generally aim to help to manage the negative reactions to tinnitus instead of elimination or alteration of the tinnitus percept, with cognitive therapeutic elements such as education, counseling, or psychological coping treatments as key elements (Cima et al., 2014). The most recent versions of psychological therapies focus on acceptance and commitment therapy, and mindfulness-based stress reduction. The rationale for dismissing more control-based elements is that their beneficence in the long run is questioned. Experimental studies show that control strategies might be counterproductive in the long run, with masking tinnitus by sound even more so as it advocates experiential avoidance, adding to unwillingness to accept tinnitus (Cima et al., 2014).

Like psychological therapies for tinnitus, sound enrichment therapies come in great variety. In a scoping review, Searchfield (2021) discusses the history of Tinnitus Sound Therapy (TST) and its treatment frameworks, together with the rationale from the perspective of behavioral neuroscience. Different types of TST include hearing aids, different forms of noise-based masking, (modifications of) Tinnitus Retraining Therapy, Music Therapy, and therapies that use different types of tinnitus matched sounds. The rationale of TST is based on three effects that may lead to adaptation to the tinnitus sound. **The presence effect** by totally or partially replacing the perception of tinnitus by perception of another sound and thus affecting bottom-up processing. **The context of sound effect** changes the perception of tinnitus because of another sound competing with, recategorizing, or defocusing attention on tinnitus. The third effect is the **reaction to sound effect** that reduces or removes negative reactions to tinnitus by exposure to sound. Like the Context of Sound Effect, the Reaction to Sound Effect is governed by cognitive processes and therefore affects top-down processing by decoupling negative emotions from tinnitus (Searchfield, 2021). In combination with interventions directed at personality or psychosocial factors, these three effects may facilitate adaptation to tinnitus.

A Cochrane review on the effect of CBT (Fuller et al., 2020) shows that it may have positive effects, but in rare cases may also have negative effects. Results show that there was evidence to support the superiority of CBT over not providing any intervention, although the evidence is limited, and effect sizes were small. When CBT is compared with audiological care (tinnitus education and rehabilitation for hearing loss), evidence was found that might indicate the superiority of CBT regarding the impact of tinnitus on quality of life. The mean difference across these studies, however, was too small to reflect a clinically important change. Furthermore, there were negligible differences between CBT and audiological care regarding improvement on measures of depression, anxiety or regarding reducing negatively biased interpretations of tinnitus. A Cochrane review on Sound Therapy (Sereda et al., 2018) concludes that there is no evidence to support the superiority of Sound Therapy over waiting list control, placebo or counseling without device. Furthermore, the authors conclude that there is insufficient evidence to support the superiority of any form of sound enrichment (hearing aid, sound generator or combination device) over the other. The quality of evidence of the reported outcomes was low, however. The results from these different Cochrane studies justify considerable professional equipoise concerning different treatment options for tinnitus. In case of professional equipoise, Shared Decision Making (SDM) is an indispensable tool for guiding patients to (the combination of) interventions that best fit their needs.

SDM is a principle enshrined in Dutch law since 2020 (Dutch Medical Treatment Agreement Act [WGBO], article 7: 448 of the Civil Code). In SDM clinicians and patients share the best available evidence for making decisions and patients are supported to consider options with the goal to reach well informed preferences (Elwyn et al., 2010, 2012). According to Stiggelbout et al. (2012) this approach should be the norm in most medical practices for several reasons. They argue that it is an ethical imperative under the widely accepted four biomedical ethical principles. These are respecting patient autonomy, beneficence (balancing benefits of treatment against risks and costs), non-maleficence (avoiding harm) and justice (distributing benefits, risks, and costs fairly). SDM is based on accepting that individual self-determination is a goal to be desired and that clinicians should support patients to achieve this goal whenever feasible (Elwyn et al., 2012). The authors acknowledge however, “… the challenges that clinicians will be navigating …” stating also that “… SDM has to be built on the core skills of good communicating skills ….” They propose a model for implementing SDM consisting of three key steps: a choice talk (conveys awareness that a choice exists), an option talk (patients are informed about treatment options in more detail) and a decision talk (patients are supported to explore ‘what matters most to them’, having become informed). For clinicians to be able to support patients in SDM, not only communication skills are needed, but also fundamental knowledge about how patients make decisions under uncertainty. This is especially true in cases of professional equipoise, that is in clinical situations where the clinician has no clear preference for a treatment option (Elwyn et al., 2000).

The aim of this paper is to develop a method to assess the accuracy and utility of patient decisions by combining concepts from the Health Belief Model (HBM) (Janz and Becker, 1984) with Signal Detection Theory as discussed in Swets et al. (2000). We will apply this method to decisions tinnitus patients make in their patient journey when choosing freely for psychosocial counseling (yes or no) and audiological care (yes or no). We will discuss how this knowledge may help in SDM.

## 2 Methods

**Figure 1:**
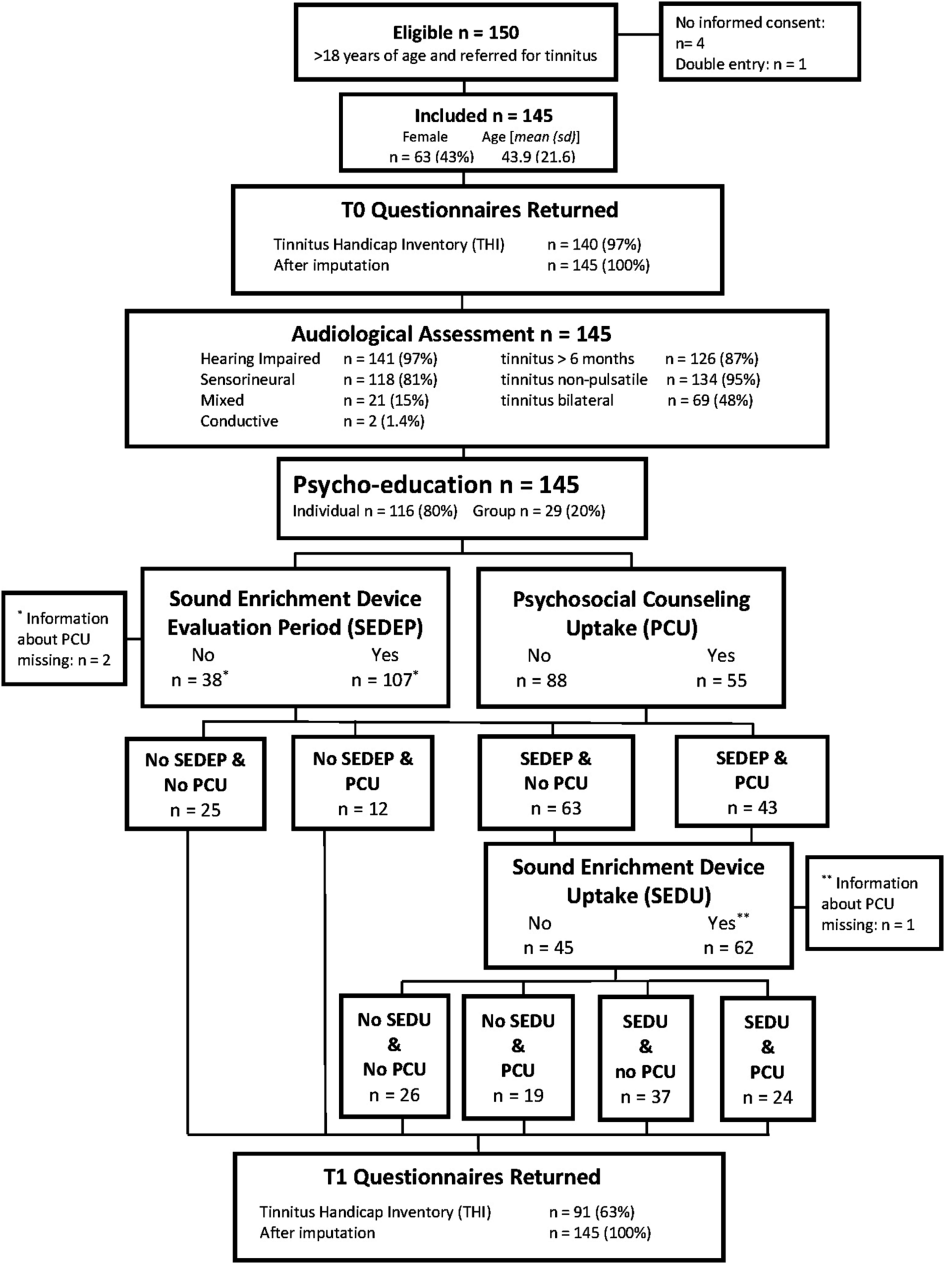
Flow chart showing experimental design and some relevant participant characteristics. See Table 3 for a summary of what type of device participants adopted as part of their treatment.

During a two-year period, all participants that were referred for tinnitus treatment to an outpatient audiological clinic in Alkmaar, the Netherlands, by a general practitioner or an otolaryngologist were asked to participate in this study until we reached the number of 150 participants. Figure 1 shows the flow chart of the study design. Permission was granted by the Medical Research Ethics Committee (MEC) Noord-Holland (M010-34). Inclusion criteria were age (18 years and older), primary referral for tinnitus care, and no current use of a hearing aid, a sound generator, or a combination device. Four participants revoked permission after inclusion and one participant turned out to be a duplicate entry, leaving 145 participants.

Clinical data of these participants was entered in a database and included the variables: air-conduction and bone conduction hearing thresholds at standard (half) octave frequencies 0.125–8 kHz in dB(HL), Tinnitus Handicap Inventory (THI) score, Glasgow Health Status Inventory score (‘The Glasgow Health Status Questionnaires Manual: 1998’, 1998), age in years, biological sex (male; female), type of hearing loss (sensorineural; conductive; mixed), tinnitus duration (≤ 2 months; 2–6 months; > 6 months), non-hearing related problems (none; problems in family life; work related problems; personality disorders), tinnitus type (tonal; noise; different), tinnitus nature (continuous; pulsating), side of tinnitus perception (left ear; right ear; central), ability to match tinnitus to external sound in a tinnitus assessment (yes; no; missing), tinnitus frequency (< 1 kHz, 1–4 kHz, ≥ 4 kHz), residual inhibition defined as the absence of tinnitus for more than 30 s after presenting masking noise for 30 s (yes; no; missing), psychoeducation counseling setting (individual session; group session), starting an evaluation period with a sound enrichment device (yes; no), purchase of a sound enrichment device after the evaluation period (yes; no), psychosocial counseling (yes; no), referring medical specialty (general practitioner; otorhinolaryngologist; missing).

All statistical analyses were conducted in Matlab R2023b Update 3 (23.2.0.2409890) unless stated differently. Continuous variables are presented as median (minimum value – maximum value) or median (1st quartile – 3rd quartile) and categorical variables by number of instances of each category (percentage). To assess differences between treatment groups, analyses of variance (ANOVAs) were run on all continuous variables with treatment options as factor, using the MATLAB function “anova” with type III sum of squares and *α* = 0.05. Also, the MATLAB function ‘crosstab’ was used for cross-tabulation of categorical variables and treatment options to test the null hypothesis that they are normally distributed across groups with the Chi-square statistic and α = 0.05. As this is an exploratory analysis without the aim to test the null hypothesis that treatment groups are homogenous for all variables, we did not adjust for multiple testing and we used Fisher’s ‘protected least squares difference’ for post-hoc testing whenever an ANOVA found a statistically significant difference (Armstrong, 2014).

### 2.1 Primary outcome measure

As primary outcome measure, we used the change in THI-score. The THI is a 25-item self-report questionnaire (Bankstahl et al., 2012; Newman et al., 1996). It quantifies the impact of tinnitus on everyday functioning on three aspects: functional, emotional, and physical. Participants can rate each item on a 3-point scale (4 = yes, 2 = sometimes, 0 = no). Total scores range from 0 to 100, which can be divided in five severity scales (0–16 = slight, 18–36 = mild, 38–56 = moderate, 58–76 = severe, 78–100 = catastrophic). After informed consent and before audiological assessment, participants filled out the baseline THI questionnaire. After tinnitus treatment participants filled out the THI questionnaire again. Response rates for the baseline questionnaire was 97% and for post-treatment questionnaire 63%. The response rate of the GHSI questionnaire was 56%. The pattern of missing data for all questionnaires was examined by using the Little MCAR test (SPSS version 24), which indicated that data were missing completely at random (*p* = 0.394).

We imputed missing data based on observed values for a particular participant and the relations observed in the data for other participants. First, we performed a linear N-way analysis of variance (ANOVA) with type III sum of squares and *α* = 0.05, using Matlab function “anovan” with baseline THI-score as dependent variable. Hearing level and age were marked as continuous predictors, all other variables as factors. All continuous predictors and factors with a *p* < 0.15 were included in a second N-way ANOVA. These were tinnitus duration (*F* = 2.736; *p* = 0.0693), non-hearing related problems (*F* = 6.190; *p* = 0.0006) and tinnitus type (*F* = 1.941; *p* = 0.1485). The second N-way ANOVA revealed significant associations (*p* < 0.05) between baseline THI-score and tinnitus duration (*F* = 3.345; *p* = 0.0383) and non-hearing related problems (*F* = 9.769; *p* < 0.0001), but not for tinnitus type (*F* = 1.2879; *p* = 0.2793). Next, we fitted a linear regression model with MATLAB-function ‘fitlm’ with independent variables tinnitus duration and non-hearing related problems and dependent variable baseline THI-score [R^2^ adjusted = 0.19; Root Mean Square Error (RMSE) = 19.8]. With the MATLAB-function ‘feval’ we imputed the missing values. The same approach was used for imputing missing values for baseline GHSI-score, but now baseline THI-score (with imputation) was included as a continuous predictor. This resulted in significant associations between baseline GHSI-score and age (*F* = 6.3274; *p* = 0.0140), non-hearing related problems (*F* = 4.6174; *p* = 0.0051); audiological care (*F* = 4.8080; *p* = 0.0314) and referring medical specialty (*F* = 3.2600; *p* = 0.0438). These variables were again used to construct a linear model for imputation of missing values (R^2^ adjusted = 0.54, RMSE = 10.2). As a last step, for imputation of missing values for post-treatment THI-score, N-way ANOVAs were performed with independent variables, all database variables, baseline THI-score (after imputation), and baseline GHSI-score (after imputation). Significant associations were found for baseline THI-score (*F* = 40.378; *p* < 0.0001), baseline GHSI-score (*F* = 19.039; *p* < 0.0001), age (*F* = 14.213; *p* = 0.0003), and biological sex (*F* = 4.6696; *p* = 0.0337). These variables were used to construct a linear model for imputation of missing variables for post-treatment THI-score (R^2^ adjusted = 0.72; RMSE = 11.3).

Several ANOVAs (Matlab function ‘anova’) were run with type III sum of squares and *α* = 0.05. First an ANOVA with change in THI-score (post treatment THI-score – baseline THI-score) as dependent variable and combinations of follow-up treatment (SEDEP and PCU, SEDEP and no PCU, no SEDEP and PCU, and no SEDEP and no PCU) as factors. Next an ANOVA was run with relative change in THI-score (post treatment THI-score – baseline THI-score)/baseline THI-score, using the same factors. Also, ANOVAs were run with change and relative change in THI-score as dependent variables and combinations of follow-up treatment after the evaluation period (no SEDEP and no PCU, no SEDEP and PCU, SEDU and no PCU, SEDU and PCU, no SEDU and no PCU, and no SEDU and PCU) as factor. As this is an exploratory analysis, we again did not adjust the significance level for multiple testing.

### 2.2 Interventions

#### 2.2.1 Audiological assessment and psychoeducation

Audiological assessment consisted of audiological history taking, pure tone audiometry, speech audiometry and tympanometry. Additionally, uncomfortable loudness was determined, and perceived tinnitus sound was assessed. Audiological equipment consisted of Interacoustics AC 40 or Interacoustics Affinity both with Telephonics TDH39 headphones and RadioEar B71 bone conductors, calibrated yearly according to ISO-389.

Tinnitus psychoeducation was provided according to standard of care in the Netherlands. In an educational session participants received information on the functioning of the ear and hearing including central auditory processing, the outcome of the audiological assessment, and the somatic and neurophysiologic causes of tinnitus. Also, the interaction of tinnitus with psychosocial functioning and physical health was discussed and related to the interaction between attention, auditory filter, and perception. Additionally, topics such as the influence of stress, emotions, and the use of medicine, drugs and alcohol were discussed as well as the follow-up therapy options (audiological care, psychosocial counseling, or a combination). All participants were offered extension of tinnitus therapy by either audiological care (sound enrichment with hearing aids with or without the option of a sound generator) and/or psychosocial counseling based on elements of CBT. Figure 1 shows for each combination of follow-up treatment options the number of participants that adopted the combination.

#### 2.2.2 Audiological care

Audiological care started with providing information about and discussing expectations of sound enrichment with hearing aids with or without the option of a sound generator, and informing participants about the procedure of an evaluation period. Participants were asked whether they would like to start an evaluation period without any obligation to purchase the device whatsoever. The decision to start an evaluation period will be referred to as sound enrichment device evaluation period (SEDEP).

The devices were provided by an independent dispenser. Participants then returned for a first audiological rehabilitation visit at the audiological center to fit the device(s). Adequate amplification was verified by real-ear measurements and sound therapy with the sound generator was provided in a separate hearing aid program either with or without amplification. We used a broadband stimulus that was adjusted in pitch and intensity according to participant’s preferences. Participants were instructed that the stimulus should be perceived as pleasant at a comfortable loudness. Participants were asked to rate the broadband stimulus loudness relative to the tinnitus percept on a scale of 0–10 (0 being inaudible and 10 being equal to the perceived loudness of the tinnitus percept). The loudness corresponding to a comfortable stimulus level had a median (first quartile – third quartile) of 5 (4–5). After this initial visit an evaluation period with the duration of two months started.

After two months, the audiologist called the participants by telephone to discuss their experience during the evaluation period. A new session for adjustment of device settings was provided whenever requested by the participant. This resulted in a prolongation of the evaluation period. When no (further) fitting adjustments where requested, participants were asked to freely decide whether to purchase the device. This will be referred to as sound enrichment device uptake (SEDU). Part of the purchase cost of the device was liable for reimbursement by national health insurance.

#### 2.2.3 Psychosocial counseling

Psychosocial counseling was provided by a social worker in 1 to 3 sessions that each lasted 60 minutes. The decision to attend the first session will be referred to as psychosocial counseling uptake (PCU). The aim of the first session was to discuss the complaints, treatment goals, and coping strategies to reduce the burden of tinnitus. History was taken to map the participant’s tinnitus burden, health status, personal factors, handling of tinnitus, and expectations regarding the information provided in psychoeducation. A second and an optional third session were dedicated to coaching the participant regarding application of coping strategies in daily life until the participant was proficient to carry on independently. If more intensive coaching or psychological counseling was needed, participants were referred to a psychologist or a specialized center for additional psychological care. No referral was needed for 125 (86.2%) participants, 5 (3.4%) participants needed referral, and for 15 (10.3%) participants data about referral was missing.

### 2.3 The health belief model

HBM is based on two well-established hypotheses from psychology and behavioral theory stating that behavior depends mainly on two variables (Janz and Becker, 1984). In the context of health-related behavior, these variables are (1) the value that a person places on avoiding an illness or (if ill) getting better and (2) the belief that a specific health action will prevent illness or (if ill) will ameliorate illness. Next, the participant’s estimate of the threat of illness is translated into two dimensions related to an illness: perceived susceptibility and perceived severity. In their review the authors report that perceived susceptibility is strongly related to preventive health behavior, while perceived severity is strongly related to sick role behavior. As tinnitus sufferers already have the condition, we assume that the behavior of patients seeking help for tinnitus is best modelled by sick role behavior.

As discussed in Janz and Becker (1984), the dimension of perceived severity relates to feelings about the seriousness of contracting a condition or leaving it untreated. These feelings may vary from person to person, and they include evaluations on both medical consequences (death, disability and pain) and possible social consequences (effects of the condition on work, family life and social relations). A review on factors influencing help seeking, hearing aid uptake, hearing aid use and satisfaction with hearing aids (Vestergaard Knudsen et al., 2010), reports that more hearing loss tends to be associated with help seeking and hearing aid uptake. An observational study on help seeking for tinnitus (Rademaker et al., 2021b) similarly reports that help seekers for tinnitus more frequently have hearing loss and score higher on tinnitus impact as measured with the Tinnitus Functional Index. Therefore, we used hearing loss (air-conduction threshold at 8 kHz at participants’ worst ear) and baseline THI-score as two independent proxies for perceived severity and thus as two independent drivers for decisions about tinnitus treatment.

The individual’s estimate of the likelihood of being able to ameliorate illness through personal action is translated into two dimensions related to action: perceived benefits and perceived costs. In the next section, these concepts will be related to the selection of an optimal decision criterion in SDT.

### 2.4 Signal detection theory

Participants were asked to make decisions of the yes-no type for the complementary treatment options, audiological care and psychosocial counseling, independently. There are two ways to improve performance on decisions of the yes-no type. One way is to enhance its accuracy, the other way is to enhance its utility (Swets et al., 2000).

#### 2.4.1 Enhancing accuracy

**Figure 2:**
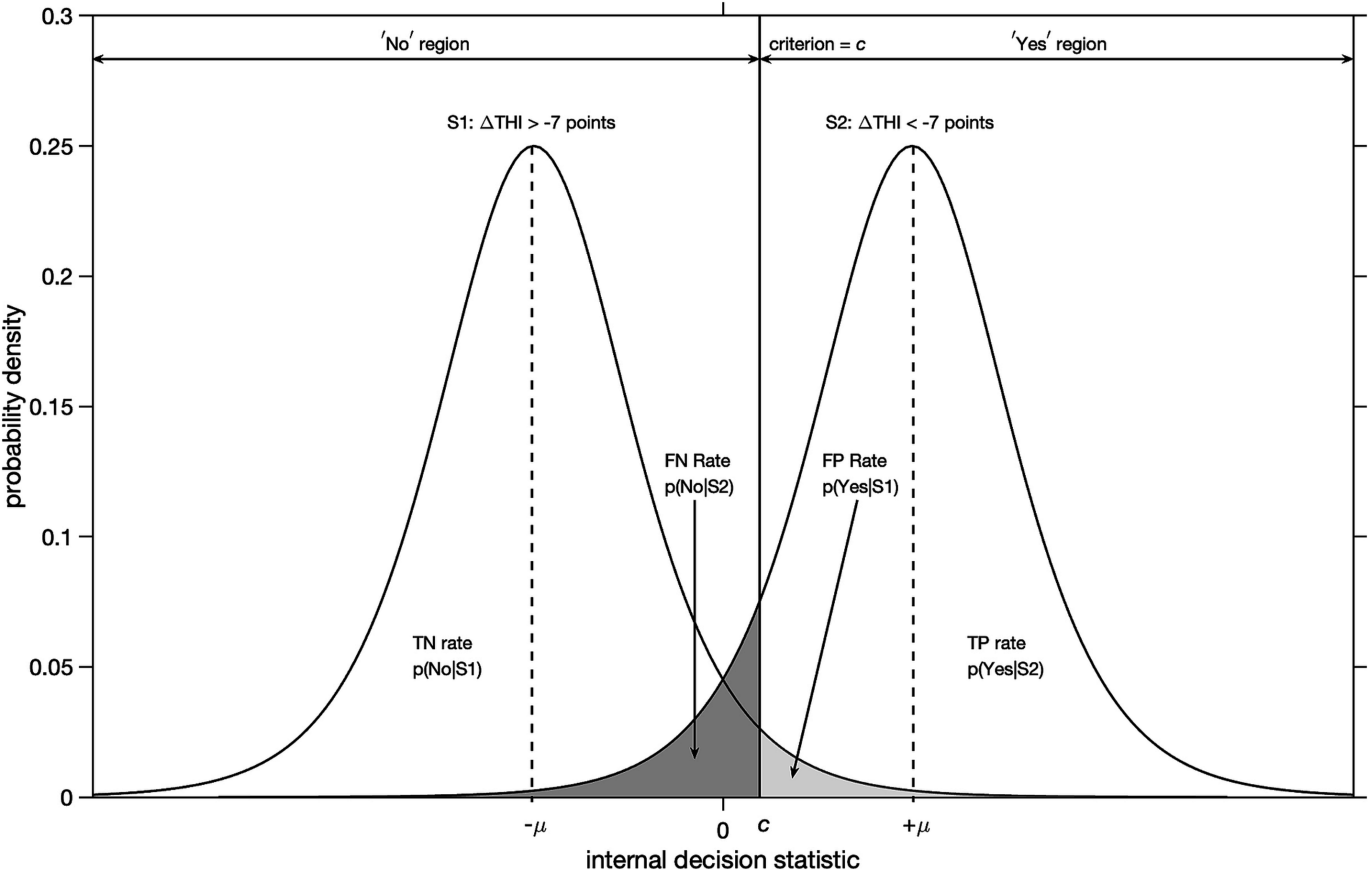
Illustration of signal detection theory for yes-no type decisions under uncertainty with logistic probability distributions. The abscissa denotes an internal decision statistic and the ordinate the probability density. A clinically important change is defined as a decrease in THI-score of more than 7 points with respect to baseline after accepting a treatment option. Distribution *S*_1_ corresponds to cases that did not experience a clinically important improvement. Distribution *S*_2_ corresponds to cases that did. The locations of modes of the distributions *S*_1_ and *S*_2_ are given by -*μ* and + *μ*, respectively. For an arbitrary value of decision criterion = c, the probabilities of true positive (TP), false positive (FP), true negative (TN) and false negative (FN) responses are given by the corresponding areas under the curves.

Enhancing the accuracy of a decision of the yes-no type is achieved by improving the ability to distinguish between the two alternatives (yes or no) and choose the correct one (Swets et al., 2000). First, we must establish a definition of a correct decision for a treatment. An obvious way to define a correct yes-decision for a treatment, is to require a clinically important improvement on a patient related outcome measure after treatment. We will call this a true positive response (TP). An incorrect yes-decision then corresponds to no clinically important change after treatment. We will call this a false positive response (FP). Likewise, we can define true negative (TN) and false negative (FN) responses. Accuracy may be increased by increasing the relative frequency of one or the other of the two types of correct decisions (TP or TN), or by decreasing the relative frequency of one or the other of the two types of errors (FP or FN) (Swets et al., 2000). For our purpose, accuracy may be enhanced by increasing the relative frequency of TP or by decreasing the relative frequency of FP, leaving TN and FN redundant as it follows from Figure 2 that the probability of TN + FP = 1 and FN + TP = 1.

We will use an improvement in THI-score of more than 7 points as yardstick for a clinically important improvement after treatment (Zeman et al., 2011). For example, a particular participant correctly decided yes (TP) for a treatment option, if for that participant the post-treatment THI-score decreased by more than 7 points relative to the baseline THI-score. Likewise, a particular participant incorrectly decided yes (FP) for a treatment option, if the post-treatment THI-score decreased by less than 7 points. This is illustrated in Figure 2.

The reason that correct and incorrect decisions occur is that the evidence available for a decision is usually ambiguous (Swets et al., 2000). For example, a higher tinnitus impact score is associated with tinnitus help seeking (Rademaker et al., 2021b), however this is merely a tendency. Patients with a low tinnitus impact score may still decide to take up treatment and patients with a high tinnitus impact score may decide not to take up treatment.

In our context, the probability of treatment success may depend on the value of an arbitrary internal decision statistic (abscissa in Figure 2). We will use statistical analyses to establish whether hearing loss and baseline THI-score are predictor variables of the internal decision statistic. Using the method presented in Macmillan and Creelman (2004), we seperated the marginal distributions for the decision to start SEDEP and the decision for PCU, respectively, from the joint distributions of the 4 combinations in Figure 1, i.e., starting SEDEP *yes*/*no* with PCU *yes*/*no*. See Supplement 1 for the details. For each combination of decision *i* (SEDEP or PCU) and driver *j* (hearing loss or baseline THI-score), we estimated with logistic regression the parameters of the cumulative distribution functions (Equation S.2.3 in Supplement 2) of the *S*_1_ population (participants that *did not* experience a clinically important improvement in THI-score) and *S*_2_ population (participants that *did* experience a clinically important improvement in THI-score). With Bayesian inference we determined the posterior probability distributions for all 4 parameter pairs *θ*_1,i,j_ = −*μ*_*i*,*j*_/*s*_*i*,*j*_ and *θ*_2,i,j_ = 1/*s*_*i*,*j*,_ of the logistic distributions corresponding to the *S*_1_ and *S*_2_ populations for SEDEP and PCU. See Supplement 3 for the details. In the remainder of this text the parameter *θ*_1_ is referred to as the intercept and *θ*_2_ as the slope.

For the data to be consistent with the assumptions of SDT, i.e., hearing loss or baseline THI-score being a driver for a particular decision, the value of the slope parameter must be different from zero. Else, the number of true positive or false positive cases would be constant with varying driver magnitude. Therefore, we calculated the Bayesian probability of the slope parameter being different from 0. This allows testing the following hypotheses:

H_0_: *θ*_2_ = 0,

H_1_: *θ*_2_ > 0.

In case of a cumulative distribution function that decreases with increasing driver magnitude *x*, the driver magnitude is transformed with *y* = *maximum value driver* – *x* to assure positive probability density functions. For rejection of the null hypothesis, a threshold of *α* = 0.01 was set for the Bayesian probabilities.

#### 2.4.2 Enhancing utility

As is argued in Swets et al. (2000), sometimes positive decisions are preferred over negative decisions. Translating this argument to our context, this can be the case if the probability of a positive treatment outcome is higher for one treatment than for another. Another reason could be selection bias. For example, if more patients in a certain clinic, for whatever reason, would benefit from a certain treatment option compared to patients in another clinic. Another reason for a preference for positive decisions may be that the benefit of being correct, when the treatment is effective, is very high. Alternatively, situations may occur that favor negative decisions. For example, when the cost of a positive decision, when the treatment is ineffective, is very high. As was stated earlier, these concepts of benefits and costs are related to HBM. Furthermore, in SDT they allow adjustment of the decision criterion to produce the best ratio of positive and negative decisions that results in maximization of utility (Swets et al., 2000).

Let us introduce the notation suggested in Swets et al. (2000). We can speak of two truth states concerning treatments, the presence or not of a clinically important improvement. Let T+ denote an effective treatment and T- and ineffective treatment. The probabilities of these truth states prior to a decision, or base rates, are denoted P(T+) and P(T-). These base rates could be obtained from randomized controlled trials, for example. Positive and negative decisions are marked as D+ and D-. Now, let us denote benefits by B and costs by C. For the benefits, that are associated with the joint occurrence of truth states and decisions that agree, we have B(T+ & D+) and B(T- & D-). For costs, that are associated with joint occurrence of truth states and decisions that disagree, we have C(T+ & D-) and C(T- and D+). The equation that resolves the optimal decision criterion that maximizes expected value is given by Swets et al. (2000),


(1) $$c_{\mathrm{optimal}}=\frac{P\left(T-\right)}{P\left(T+\right)}\frac{\left\{B\left(T-\&D-\right)+C\left(T-\&D+\right)\right\}}{\left\{B\left(T+\&D+\right)+C\left(T+\&D-\right)\right\}}$$

In case base rates of both truth states are equal and all costs and benefits are equal, *c_optimal_* is called unbiased and corresponds to the driver value at which the likelihood of a TP response equals the likelihood of a FP response, i.e., the likelihood ratio of TP and FP is 1. A lenient decision criterion corresponds to a likelihood ratio < 1 and a strict decision criterion to a likelihood ration >1.

## 3 Results

Figure 1 shows the flow chart of the study design. For 2 participants information about PCU was missing, one had chosen SEDEP and the other had not, leaving 143 participants with information on choices for starting SEDEP and PCU. For 1 participant, that had chosen to purchase the device (SEDU), information about PCU was missing. This leaves 106 participants with information on choices for the combination of SEDU and PCU.

Table 1 shows non-tinnitus percept related variables grouped per treatment. The ANOVA of age versus treatment options showed statistically significant differences in means between groups. Post-hoc analysis showed that the group with only PCU was younger than the other groups, mean difference (95% confidence interval [CI]) with SEDEP and no PCU is 15 (8–23) years, *p* = 0.0001; with SEDEP and PCU is 14 (6–22) years, *p* = 0.0008; with no SEDEP and no PCU is 10 (2–19) years, *p* = 0.0197; with PCU missing is 22 (4–40) years, *p* = 0.0178. The ANOVA of hearing loss versus treatment options also showed statistically significant differences between groups. The group with only SEDEP had more hearing loss than the groups with only PCU, and the group with no SEDEP and no PCU. Mean difference (95% CI) with only PCU is 24 (8–41) dB(HL), *p* = 0.0037; with no SEDEP and no PCU is 18 (5–30) dB(HL), *p* = 0.0049. The group with no SEDEP and no PCU had less hearing loss than the group with SEDEP and PCU [13 (26–0) dB(HL), *p* = 0.0443]. The group with SEDEP and PCU had more hearing loss than the group with only PCU [20 (3–37) dB(HL); *p* = 0.0205]. From the perspective of HBM, this suggests that more hearing loss is associated with a higher probability to start SEDEP. These results might also point at an interaction between hearing loss and age, where older participants tend to have more hearing loss and may therefore more often have chosen SEDEP. A bias of older participants against PCU cannot be ruled out however.

**Table 1:** General characteristics of the cohort.

		No SEDEP & no PCU	No SEDEP & PCU	SEDEP & no PCU	SEDEP & PCU	PCU missing	*p*
Participants (*n* = 145)		25 (17)	12 (8)	63 (43)	43 (30)	2 (1)	
Age (years)		55 (33–75)	48 (18–57)	60 (26–86)	58 (31–84)	66 (49–83)	0.0017^*^
Hearing loss [dB(HL)]		45 (10–100)	35 (5–95)	65 (15–115)	60 (10–115)	60 (20–100)	0.0082^*^
THI-score (points)	Baseline complete case	34 (8–84)	58 (28–76)	38 (6–80)	48 (8–94)	36 (36–36)	0.0150^*^
	Baseline imputed	34 (8–84)	58 (28–76)	38 (6–80)	48 (8–94)	37 (36–38)	0.0157^*^
	Post-treatment complete case	32 (4–72)	40 (20–70)	36 (4–70)	37 (2–94)	-	0.3645
	Post-treatment imputed	25 (3–72)	41 (20–70)	34 (4–70)	35 (2–94)	27 (27–27)	0.1475
GHSI-score (points)	Baseline complete case	65 (49–90)	54 (36–71)	54 (22–86)	53 (16–90)	–	0.1019
	Baseline imputed	65 (44–90)	54 (36–71)	54 (22–86)	53 (16–90)	58 (49–66)	0.0390^*^
Biological sex	Male	11 (44)	9 (75)	34 (54)	27 (63)	1 (50)	0.3858
	Female	14 (56)	3 (25)	29 (46)	16 (37)	1 (50)	
Type of hearing loss	Sensorineural	19 (76)	9 (75)	50 (79)	39 (91)	1 (50)	0.0140^*^
	Conductive	0 (0)	1 (8)	1 (2)	0 (0)	0 (0)	
	Mixed	4 (16)	0 (0)	12 (19)	4 (9)	1 (50)	
	Missing	2 (8)	2 (17)	0 (0)	0 (0)	0 (0)	
Refering medical specialty	General practitioner	9 (36)	0 (0)	21 (33)	5 (12)	0 (0)	<0.0001^*^
	Otorhinolaryn-gologist	12 (48)	4 (33)	40 (63)	12 (28)	2 (100)	
	Missing	4 (16)	8 (67)	2 (3)	26 (60)	0 (0)	
Non-hearing related problems	None	18 (72)	3 (25)	50 (79)	24 (56)	1 (50)	0.0375^*^
	Family live	6 (24)	5 (42)	9 (14)	14 (33)	1 (50)	
	Work	1 (4)	1 (8)	1 (2)	2 (5)	0 (0)	
	Personality disorders	0 (0)	3 (25)	3 (5)	3 (7)	0 (0)	

Continuous variables are presented as median (minimum–maximum) and categorical variables as number of instances of each value (percentage). ^*^p < 0.05.

The ANOVAs of baseline THI-score versus treatment option showed statistically significant differences in group means both for complete case analysis (all participants with missing data removed) and with imputation. The group with only SEDEP had a lower baseline THI-score than the groups with SEDEP and PCU [mean difference (95% CI) complete case 10 (2–19) points, *p =* 0.0157; imputation 10 (2–18) points, *p* = 0.0157], and only PCU [complete case 16 (3–29) points, *p* = 0.0179; imputation 16 (3–29) points, *p* = 0.0159]. The group with SEDEP and PCU had a higher baseline THI-score than the group with no SEDEP and no PCU [complete case 14 (3–25) points, *p* = 0.0133; imputation 13 (2–23) points, *p* = 0.0171]. The group with no SEDEP and no PCU had a lower baseline THI-score than the group with PCU only [complete case 20 (5–35) points, *p* = 0.0111; imputation 19 (4–33) points, *p* = 0.0122]. These results show a general trend of higher baseline THI-scores for participants that choose PCU. From the perspective of HBM, this suggests that a larger perceived tinnitus impact leads to more uptake of psychosocial counseling. Also, the effect of imputation on group means is small. There were no differences between groups concerning post-treatment THI-scores, both for complete case analysis and with imputation.

The ANOVA for GHSI-score versus treatment only showed statistically significant differences between groups with imputation, but not for complete case analysis. Imputation thus has a significant impact on GHSI-score, possibly as result of the low response rate for this questionnaire (56%). The group with no SEDEP and no PCU had a higher GHSI-score (after imputation) than the groups with SEDEP and PCU [mean difference (95% CI) is 11 (4–17) points; *p* = 0.0025], SEDEP and no PCU [8 (2–15) points; *p* = 0.0103], and no SEDEP and PCU [10 (0–19); *p* = 0.0420]. This result is expected as a higher general quality of live score would be associated, in terms of HBM, with a decreased incentive to take up a health intervention.

Participants that chose SEDEP and PCU seemed to have sensorineural hearing loss more often than the other groups, a finding we cannot explain. The group with only SEDEP showed most referrals by an otolaryngologist. This group also showed somewhat more mixed hearing loss, which may be understood from a possible higher prevalence of middle ear disease in this population. Non-hearing related problems are more prevalent in the groups that chose only PCU, and SEDEP and PCU, which can be expected. Table 2 shows tinnitus percept related variables grouped by treatment option. No statistically significant differences were found between groups.

**Table 2:** Tinnitus characteristics of the cohort.

		No SEDEP & no PCU (*n* = 25)	no SEDEP & PCU (*n* = 12)	SEDEP & no PCU (*n* = 63)	SEDEP & PCU (*n* = 43)	PCU missing (*n* = 2)	*p*
Tinnitus duration	2 months or less	0 (0)	1 (8)	0 (0)	3 (7)	0 (0)	0.0553
	2–6 months	3 (12)	4 (33)	5 (8)	3 (7)	0 (0)	
	More than 6 months	22 (88)	7 (58)	58 (92)	37 (86)	2 (100)	
Tinnitus side	Right ear	4 (16)	1 (8)	21 (33)	10 (23)	2 (100)	0.1068
	Left ear	8 (32)	2 (17)	17 (27)	11 (26)	0 (0)	
	Central	13 (52)	9 (75)	25 (40)	22 (51)	0 (0)	
Tinnitus type	Tonal	6 (24)	3 (25)	19 (30)	13 (30)	0 (0)	0.8943
	Noise	13 (52)	5 (42)	29 (46)	18 (42)	2 (100)	
	Different	6 (24)	4 (33)	15 (24)	12 (28)	0 (0)	
Tinnitus nature	Continuous	23 (92)	12 (100)	61 (97)	39 (91)	2 (100)	0.5648
	Pulsatile	2 (8)	0 (0)	2 (3)	4 (9)	0 (0)	
Tinnitus matchability	Yes	22 (88)	8 (67)	45 (71)	30 (70)	2 (100)	0.2703
	No	3 (12)	4 (33)	11 (17)	13 (30)	0 (0)	
	Missing	0 (0)	0 (0)	7 (11)	0 (0)	0 (0)	
Tinnitus frequency	1 kHz or less	3 (12)	1 (8)	2 (3)	1 (2)	0 (0)	0.5798
	1–4 kHz	6 (24)	3 (25)	14 (22)	6 (14)	0 (0)	
	more than 4 kHz	12 (48)	7 (58)	30 (48)	27 (63)	2 (100)	
	missing	4 (16)	1 (8)	17 (27)	9 (21)	0 (0)	
Residual inhibition	Yes	11 (44)	6 (50)	18 (29)	21 (49)	0 (0)	0.5013
	No	9 (36)	4 (33)	26 (41)	12 (28)	1 (50)	
	Missing	5 (20)	2 (17)	19 (30)	10 (23)	1 (50)	

Categorical variables are represented as number of instances of each value (percentage).

Table 3 shows a summary of what devices participants adopted as part of their treatment. We found statistically significant differences in adopted devices between groups. Most participants started SEDEP with a combination device. However, in the group of participants that declined to purchase a device after an evaluation period (SEDU no), 60% had tried a combination device. In the group that purchased a device (SEDU yes), 42% adopted a combination device. In the SEDU no group 13% of the participants declined amplification, and in the SEDU yes group 37% adopted it after an evaluation period. This may be the result of the large proportion of participants with hearing loss in our cohort. Hearing loss was defined as any hearing threshold >15 dB(HL), however. Therefore, a considerable number of participants with hearing loss would normally not have been serviced with hearing aids. For broadband masking, the proportion of participants that accepted or declined the device were about equal (21% versus 27%). For all devices, only part of the costs of purchase were liable for reimbursement by medical insurance. This may have influenced decision making concerning SEDU.

**Table 3:** Summary of what devices participants adopted as part of their treatment.

	Amplification	Broadband masking	Combination	*p*
SEDEP yes (*n* = 107)	29 (27)	25 (23)	53 (50)	
SEDU yes (*n* = 62)	23 (37)	13 (21)	26 (42)	< 0.0234
SEDU no (*n* = 45)	6 (13)	12 (27)	27 (60)	

Categorical variables are represented as number of instances of each value (percentage).

Broadband masking may be dominated by the Context of Sound Effect and Reaction to Sound effect as the masking level was set below the sensation level of the tinnitus percept, which is generally within 5–15 dB of the hearing threshold. At this low level, a substantial Presence of Sound effect might be unlikely. The combination of amplification and broadband masking is more likely to be dominated by all three effects. Our finding, that a combination of amplification and broadband masking is most often rejected (60%), might indicate that a combination of a bottom-up effect (Presence of Sound Effect) and top-down effect (Context of Sound Effect and/or Reaction to Sound effect) is less effective. Consequently, this also might suggest that the Presence of Sound Effect is the more prominent effect when only amplification is provided, as the rejection rate for amplification only is lower than for combination devices.

**Figure 3:**
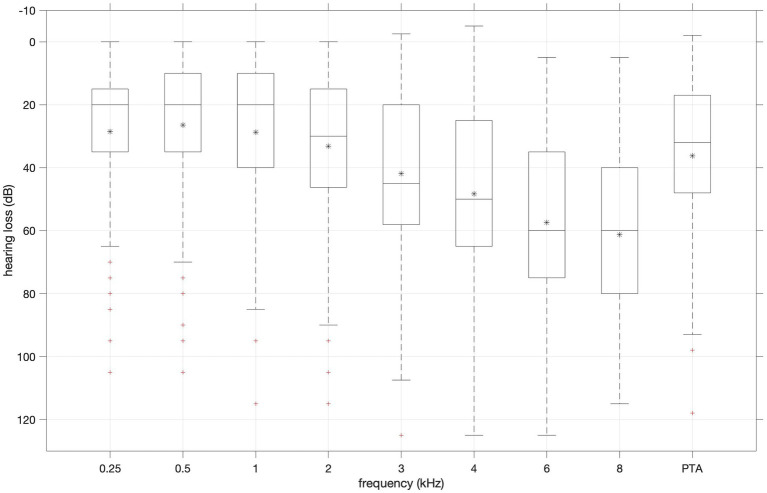
Distribution of hearing loss values for (half) octave frequencies 0.25–8 kHz and pure tone average (PTA) of 1, 2 and 4 kHz. Boxplots show median (horizontal line in the box), 25th (top edge of the box) and 75th percentiles (bottom edge of the box). Whiskers mark nonoutlier minimum and maximum values. Crosses mark outliers defined as values more than 1.5 times the interquartile range away from the boxes.

Figure 3 shows a box plot of hearing losses for (half) octave frequencies 0.25–8 kHz and pure tone average (PTA) of 1, 2 and 4 kHz. Median hearing loss increased with increasing frequency. Hearing loss at 8 kHz was chosen as proxy for perceived severity as it spans a large range (5–115 dB), and median is close to mean.

### 3.1 Primary outcome measure

An ANOVA with change in THI-score as dependent variable and combinations of follow-up treatment (SEDEP and PCU, SEDEP and no PCU, no SEDEP and PCU, and no SEDEP and no PCU, see Figure 1) as factor showed no treatment effect with imputation (*F* = 2.12, *p* = 0.1004) nor with complete case analysis (*F* = 1.939, *p* = 0.1292). Analysis of residuals showed moderate correlation of the residuals with baseline THI-score (imputation *ρ* = −0.35, *p* < 0.0001; complete case analysis *ρ* = −0.26, *p* < 0.0125) and age (imputation *ρ* = −0.29, *p* = 0.0004; complete case analysis *ρ* = −0.30, *p* = 0.0041). Therefore, we repeated an ANOVA with relative change in THI-score (i.e., change in THI-score normalized to baseline THI-score) as dependent variable and follow-up treatment as factor. Again, no effect of treatment was found with imputation (*F* = 1.886, *p* = 0.1347) nor with complete case analysis (*F* = 0.8685, *p* = 0.4607), but now we found no correlation between residuals and baseline THI-score (imputation *p* = 0.3424; complete case *p* = 0.7738) or age (imputation *p* = 0.3782; complete case *p* = 0.7154).

Table 4 shows group means (95% confidence interval) for change in THI-score grouped by treatment. The changes in THI-score for only SEDEP and only PCU, both with imputation and complete case analysis, are in line with the change after 12 months of −5.22 points for standard audiological care and − 12.8 points for specialized care found in Cima et al. (2012). This study reports on audiological care for tinnitus in the Netherlands that is very similar to our study. The specialized care, however, was much more extensive than the psychosocial counseling in our study.

**Table 4:** Group means (95% confidence interval) of the primary outcome measure for different treatment groups for the imputed data set and data set with complete cases.

		No SEDEP & no PCU	No SEDEP & PCU	SEDEP & no PCU	SEDEP & PCU
	Imputed	(*n* = 25)	(*n* = 12)	(*n* = 63)	(*n* = 43)
	Complete case	(*n* = 9)	(*n* = 11)	(*n* = 43)	(*n* = 28)
change in THI-score	Imputed	−8.56 (−13.5 to −3.59)	−13.4 (−21.1 – −5.76)	−5.72 (−8.75 – −2.68)	−10.4 (−14.1 – −6.68)
	Complete case	−7.33 (−18.0–3.34)	−12.5 (−21.8 – −3.12)	−5.35 (−9.62 – −1.07)	−12.8 (−18.2 – −7.40)

Overall median (first quartile – third quartile) change in THI-score in our cohort was −7.35 (−16–0) points with imputation and − 8.00 (−16–0) for complete case analysis. The maximum improvement was −56 points and the maximum deterioration 28 points for both imputation and complete case analysis. This demonstrates the large variance in individual outcomes underlying the relatively small median treatment effect. Remember that a clinically important change in THI-score is 7 points. For imputation, the overall median (first quartile – third quartile) relative change was −18% (−35–0%), and for complete case analysis −13% (−40–0%). For both imputation and complete case analysis we found a maximum relative improvement of −89% and a maximum deterioration of 67%.

**Figure 4:**
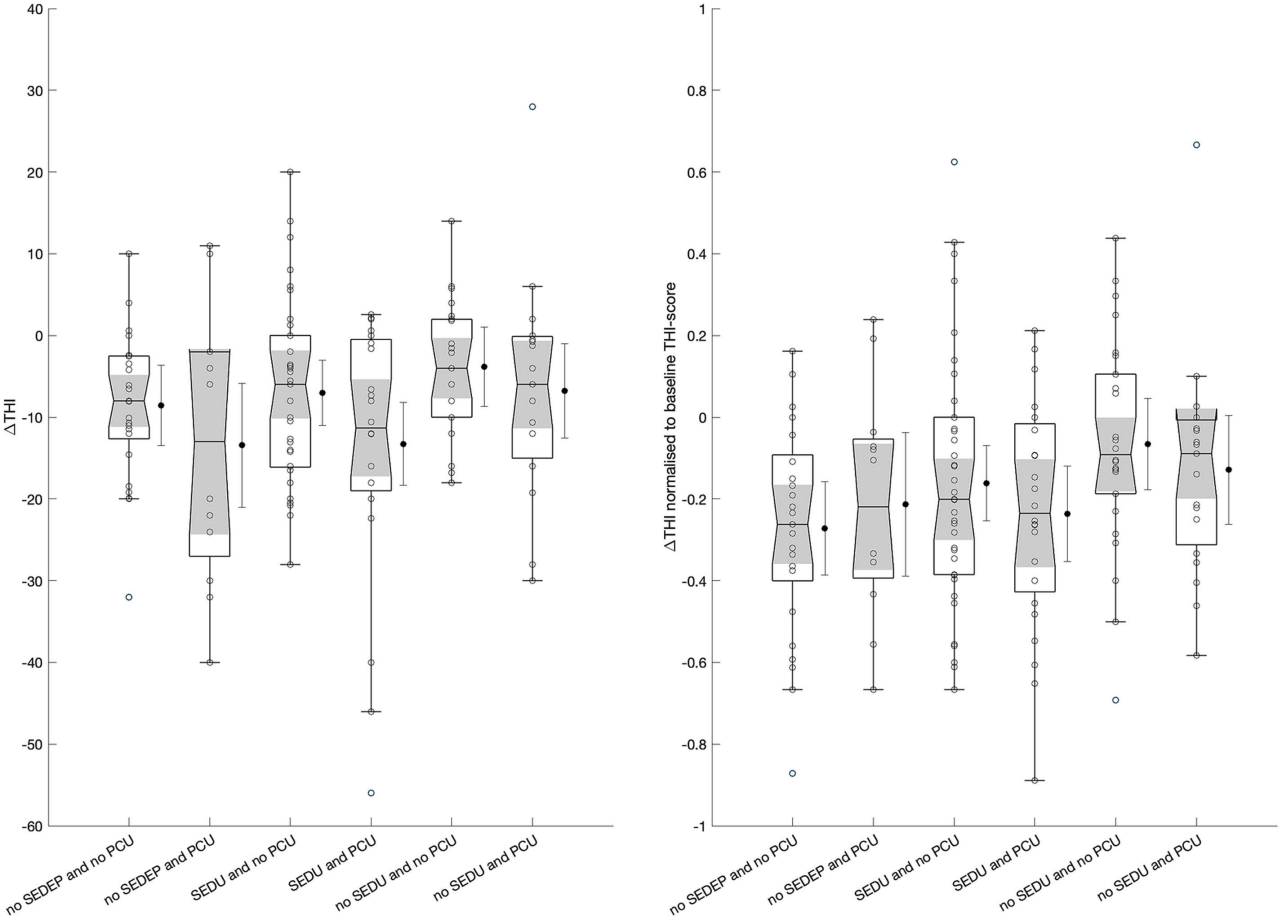
Boxplots of change in THI-score (left) and relative change in THI-score (right) for the different treatment options. Boxplots show median (horizontal line), 25th and 75th percentiles, and outliers (circles) defined as values more than 1.5 times the interquartile range away from the boxes. The whiskers mark nonoutlier minimum and maximum values. The notches (shade areas) display the variability of the medians, with boxes having non-overlapping notches having different medians at the 5% significance level. The black dots display the treatment means with wishers indicating the 95% confidence intervals. SEDEP = starting an Sound Enrichment Device Evaluation Period; SEDU = Sound Enrichment Device Uptake; PCU = Psychosocial Counseling Uptake.

An ANOVA with change in THI-score (with imputation) as dependent variable and combinations of follow-up treatment after the evaluation period (no SEDEP and no PCU, no SEDEP and PCU, SEDU and no PCU, SEDU and PCU, no SEDU and no PCU, and no SEDU and PCU, see Figure 1) as factor showed no treatment effect (*F* = 2.141, *p* = 0.0642) although it is close to statistical significance at the 5% significance level. However, analysis of residuals again showed correlation of the residuals with baseline THI-score (*ρ* = −0.36, *p* < 0.0001) and age (*ρ* = −0.27, *p* = 0.0009). Therefore, we repeated an ANOVA with relative change in THI-score as dependent variable and follow-up treatment as factor. Again, no effect of treatment was found (*F* = 1.831, *p* = 0.1107), and no correlation between residuals and baseline THI-score (*p* = 0.3505) or age (*p* = 0.4355). Figure 4 shows the boxplots of the ANOVA results for change (left panel) and relative change (right panel) in THI-score for all treatment options.

### 3.2 Signal detection theory

**Figure 5:**
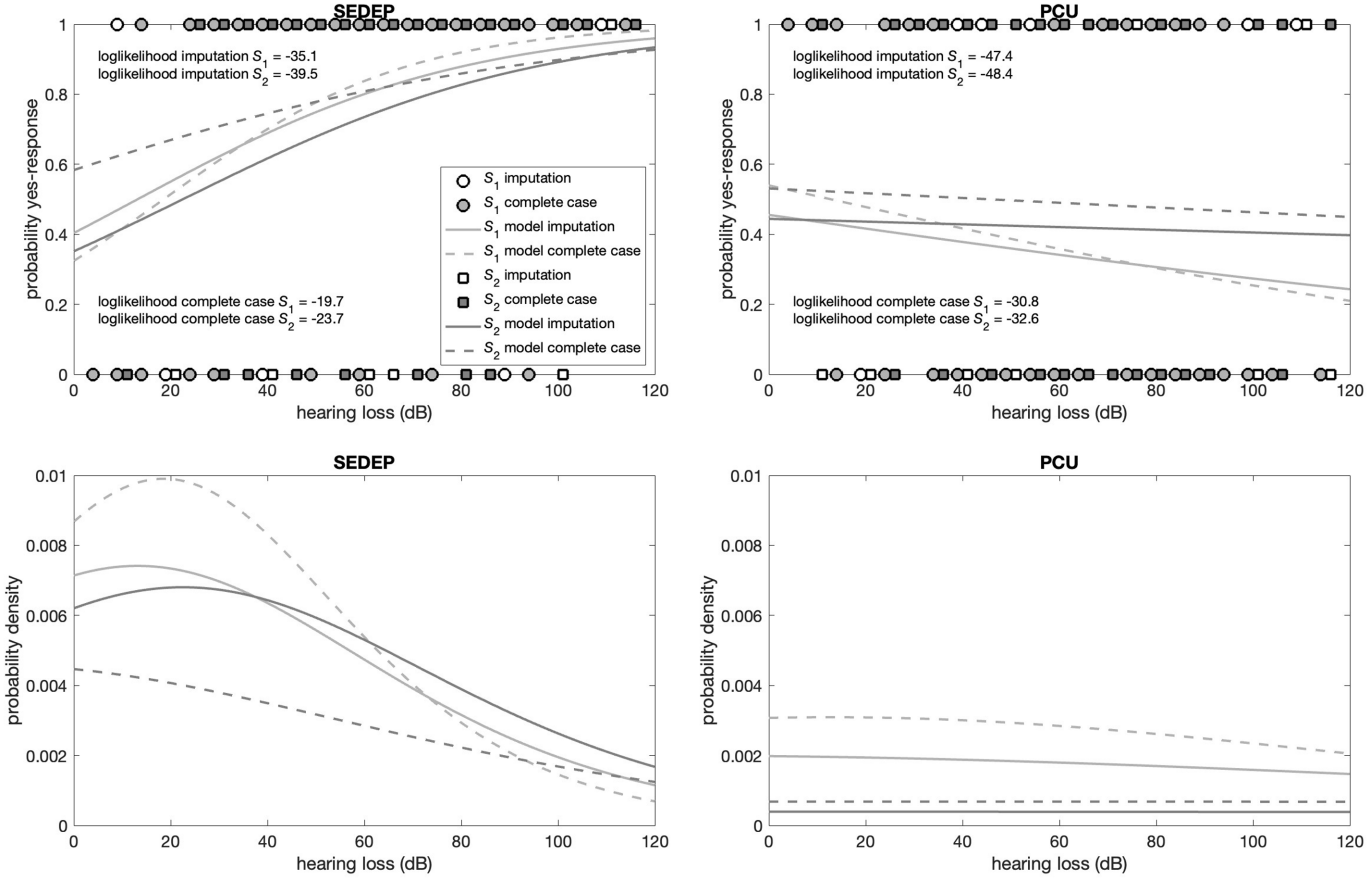
Top panels show the decisions (filled symbols) of the participants with hearing loss as driver. The left panel shows the results for the decision to start an evaluation period (SEDEP) and the right panel for taking up psychosocial counseling (PCU). Dark grey squares mark the decisions from the population that did experience a clinically important change (S2) and light grey circles mark decisions from the population that did not experience a clinically important change (S1), with 0 representing a no-decision and 1 a yes-decision. Open symbols mark imputed values. The solid lines mark the fitted cumulative distribution functions with imputation and the dashed lines for complete case analysis. The bottom panels present the corresponding probability density functions.

**Figure 6:**
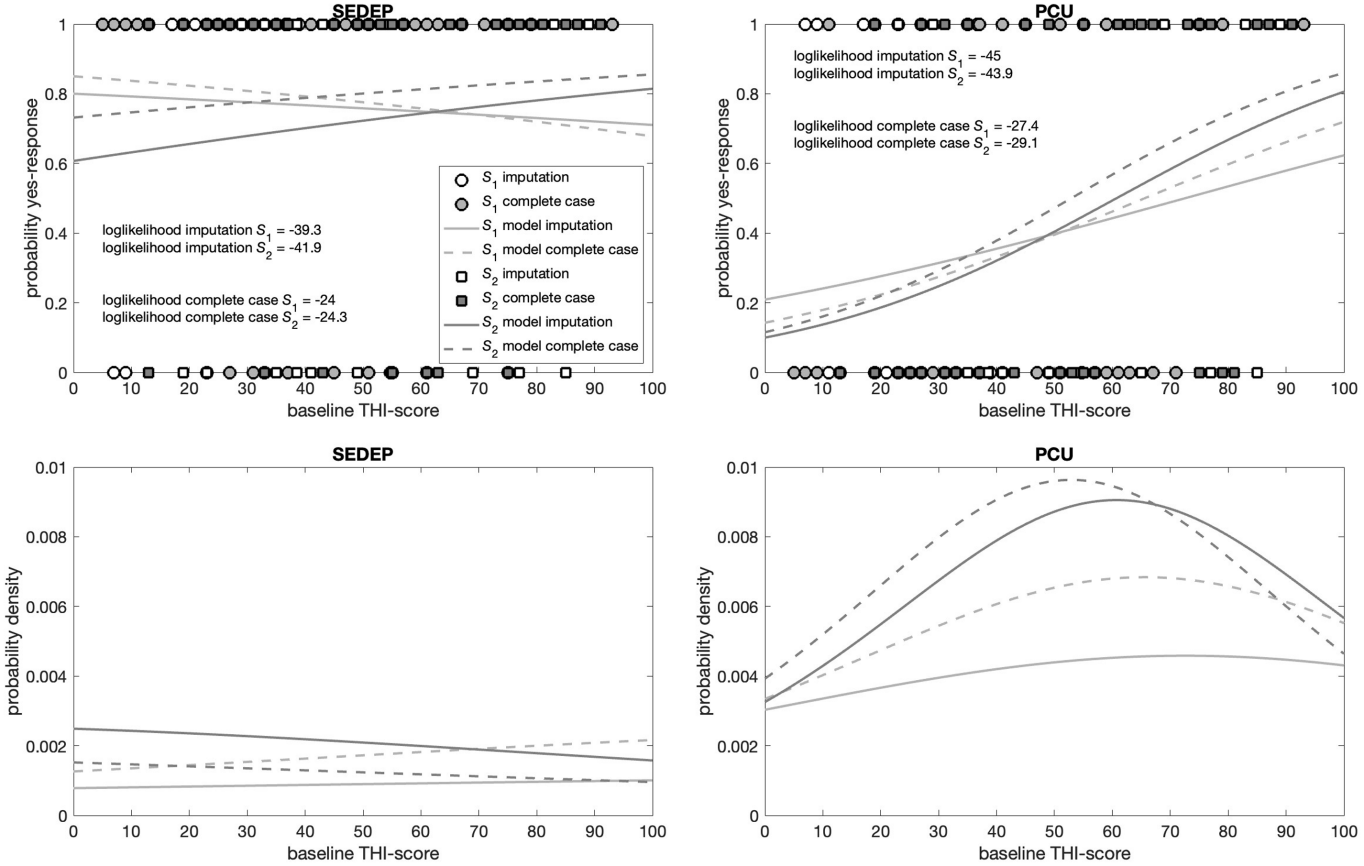
Top panels show the decisions (filled symbols) of the participants with baseline THI-score as driver. The left panel shows the results for the decision to start an evaluation period (SEDEP) and the right panel for taking up psychosocial counseling (PCU). Dark grey squares mark the decisions from the population that did experience a clinically important change (S2) and light grey circles mark decisions from the population that did not experience a clinically important change (S1), with 0 representing a no-decision and 1 a yes-decision. Open symbols mark imputed values. The solid lines mark the fitted cumulative distribution functions with imputation and the dashed lines for complete case analysis. The bottom panels present the corresponding probability density functions.

The top panels in Figures 5, 6 show the responses (no = 0 and yes = 1) of the participants plotted against hearing loss and baseline THI-score, respectively. It may be appreciated that the estimated cumulative distribution functions for the combination of SEDEP and hearing loss (top left panel in Figure 5) and the combination of PCU and baseline THI-score (top right panel in Figure 6) resembled a sigmoid curve, whereas for the combinations of SEDEP and baseline THI-score (top left panel in Figure 6) and PCU and hearing loss (top right panel in Figure 5) the curves appeared to be more linear. This is reflected in the bottom panels showing the corresponding probability density functions. Those for the combinations of SEDEP and hearing loss, and PCU and baseline THI-score showed probability density functions with a clearly identifiable mode. The others showed approximately constant probability density functions. It is clear from Figures 5, 6 that imputation impacted model fitting most for SEDEP and hearing loss (see also Supplement 3 for a discussion of the effect of imputation). The cumulative distribution and probability density functions of populations *S*_1_ and *S*_2_ were much more alike after imputation. For PCU and baseline THI-score, the impact of imputation was largest for population *S*_1_.

Supplementary Tables S.3.1, S.3.2 present the output of the Bayesian fitting procedure with imputation and for complete case analysis, respectively. All Bayesian confidence intervals (BCIs) were very close to 95%, indicating that the lower and upper bounds approximated 95% confidence intervals. For hearing loss and SEDEP, the probability of the slope having a value greater than zero was significant at the 0.01 level for both populations *S*_1_ and *S*_2_, regardless of imputation. This indicates that the data is consistent with hearing loss being a driver of the decision to start SEDEP. The same holds for baseline THI-score and PCU. Furthermore, the maximum likelihood estimates (MLEs) of the slopes, which are proportional to the inverse of the variance of the distributions of populations *S*_1_ and *S*_2_, differed most for baseline THI-score and PCU and were almost equal for hearing loss and SEDEP with imputation. For complete case analysis the slopes differed considerably for hearing loss and SEDEP.

For hearing loss and PCU the slope of population *S*_2_ was not statistically significantly different from zero at the 0.01 level, regardless of imputation. Also, the MLEs of the slopes of populations *S*_1_ and *S*_2_ were orders of magnitude smaller than the MLEs of the slopes for hearing loss and SEDEP, and baseline THI-score and PCU. For baseline THI-score and SEDEP, the slopes of populations *S*_1_ and *S*_2_ were both not statistically significantly different from zero at the 0.01 level. Again, the MLEs of the slopes were orders of magnitude smaller than the MLEs of the slopes for hearing loss and SEDEP, and baseline THI-score and PCU. This indicates that the data is not consistent with hearing loss being a driver for decisions about PCU, and baseline THI-score being a driver for decisions about SEDEP.

**Figure 7:**
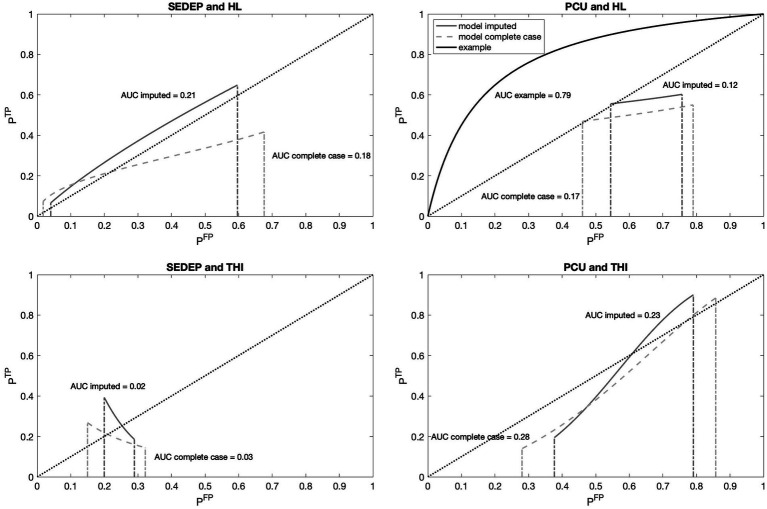
Receiver operating characteristic (ROC) curves for decisions SEDEP and PCU with hearing loss (HL) and baseline THI-scores (THI) as drivers. Abscissas show the probability of a false positive (FP) decision and ordinates show the probability of a true positive (TP) decision. The dashed diagonal lines represent the identity curve corresponding to chance performance. The solid gray lines show the model results with imputation and the dashed gray lines for complete case analysis. The solid black line in the top right panel shows, as an example, the ROC curve that corresponds to an equal variance signal detection model as presented in Figure 2. For each ROC curve the corresponding Area Under the Curve (AUC) is displayed.

Figure 7 shows the receiver operating characteristic (ROC) curves for the four combinations of drivers and decisions. As an example, the ROC curve corresponding to the case of equal variance (see Figure 2) is shown in the top right panel. The normalized distance between the modes (2 *μ*/*s*) was set at an arbitrary value of 2. The resultant area under the curve (AUC), which is a measure of the accuracy of the decision process, is also displayed in the figure. It is clear from Figure 7, that the ROC curves based on the data (both with imputation and complete case analysis) were very different from the example curve. The only exception was the curve for SEDEP and hearing loss after imputation. Remember that the slopes of populations *S*_1_ and *S*_2_ for this combination were almost equal.

Focusing on the difference between SEDEP and hearing loss (top left panel), and PCU and baseline THI-score (bottom right panel), it may also be noted that, with imputation, the curve corresponding to SEDEP and hearing loss was completely located above the identity line and the curve corresponding to PCU and baseline THI-score was partly located below the identity line and partly above it. The area above the identity line corresponds to better than chance performance and the area below that line corresponds to worse than chance performance. Also, the AUCs of both decisions were 0.21 for SEDEP and hearing loss and 0.23 for PCU and baseline THI-score, suggesting comparable low accuracy (chance performance would correspond to an AUC of 0.5). However, this low accuracy also reflects the observation that the ROC curves did not start in the bottom left corner (0,0) and did not end in the top right corner (1,1), as did the example curve. Consequently, an ROC analysis assuming equal variances of the distributions of populations *S*_1_ and *S*_2_ would lead, in our case of unequal variances for PCU and baseline THI-score, to misinterpretation of the results.

**Figure 8:**
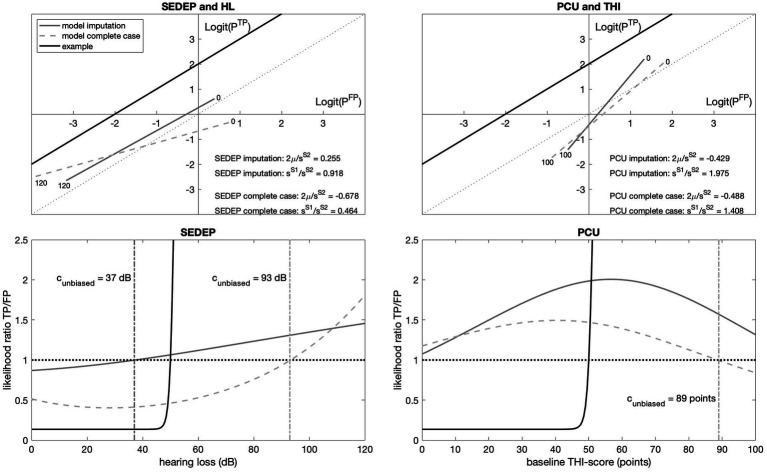
The top panels show the logit transformed receiver operating characteristic (ROC) curves for the decision to start an evaluation period (SEDEP) with hearing loss (HL) as decision driver (left) and psychosocial counseling uptake (PCU) and baseline THI-score (THI) as decision driver (right). The abscissa and ordinate denote the logit of the probability of a false positive (FP) decision and a true positive (TP) decision, respectively. The solid grey lines represent the model predictions with Equations S.2.5a, S.2.5b (see Supplement 2) with imputation and the dashed grey lines for complete case analysis. The numbers at the beginning and ending of the ROC curves denote the minimum and maximum value of the drivers, indicating the trajectory of the ROC curve. The dotted black line shows the identity line with slope coefficient = 1, corresponding to chance performance. The black solid line corresponds to the example in Figure 2. The bottom panels show the corresponding likelihood ratio of TP and FP as a function of hearing loss (left) or baseline THI-score (right). The horizontal dotted line marks a likelihood ratio of 1 and the vertical dashed-dotted lines mark the location of the unbiased decision criterion *c*_unbiased_ (i.e., the driver magnitude at which the likelihood ratio equals 1).

To visualize the effect of unequal variances, we plotted the logit transformation of the probability of true positives against the logit transformation of the probability of false positives (DeCarlo, 1998; DeCarlo, 2010). The top panels in Figure 8 show the results. Results from a decision task with equal variances of populations *S*_1_ and *S*_2_ would result in a line that is parallel to the identity line and that would cross the ordinate at a value that equals 2 *μ*/*s*, as is again illustrated by the example (corresponding to Figure 2). The parameter 2 *μ*/*s* represents the accuracy of the decision process and equals the normalized distance between the modes of *S*_1_ and *S*_2_. Our results showed that for SEDEP and hearing loss (top left panel in Figure 8) with imputation, the slope of the ROC curve was 0.918 and the line crossed the ordinate at 0.255. For complete case analysis, the ROC curve for SEDEP and hearing loss showed quite a different trajectory, with a slope of 0.464 and intersection of the ordinate at −0.678. For PCU and baseline THI-scores (top left panel in Figure 8) the results for imputation and complete case analysis were more alike. Both trajectories had almost equal intersection points of the ordinate.

Comparing the trajectories for SEDEP and PCU, it can be noted that for SEDEP changing the decision criteria from strict (at 120 dB) to lenient (at 0 dB), the logit of the probability of a TP response increased with almost the same amount as the logit of the probability of a FP response. In the case of PCU, the logit of the probability of a TP response increased with almost twice the amount of the logit increase of the probability of a FP response. This seems intuitively in line with results from the literature that state that CBT more effectively reduces the impact of tinnitus on quality of live, as measured with the TFI, when compared to audiological care (Fuller et al., 2020). This finding for PCU also holds for the results obtained with complete case analysis. The largest impact of imputation was on the trajectory of SEDEP, where for complete case analysis the increase of the logit of the probability of a TP response was decreased to roughly half the increase of the logit of the probability of a FP response, compared to imputation.

The bottom row panels in Figure 8 show the (posterior) likelihood ratio of true positive cases and false positive cases as a function of decision driver. The decision criterion for SEDEP (left panel) changes non-linearly from strict (a likelihood ratio < 1) for driver values below the unbiased decision criterion to lenient (a likelihood ratio > 1) for driver values above the unbiased decision criterion. With imputation the unbiased decision criterion matched a driver value of 37 dB(HL). With imputation the decision criterion for PCU is always strict, indicating that all participants had a higher likelihood of improvement when choosing psychosocial counseling, irrespective of their baseline THI-score. A maximum likelihood ratio of 2.01 was found for a baseline THI-score of 58 points. For lower baseline THI-scores the likelihood of a true positive outcome decreased, which can be understood by appreciating that less improvement is likely for lower initial tinnitus impact. For higher initial tinnitus impact, on the other hand, the intervention offered may be insufficient. Again, it may be noted that these results are in line with Fuller et al. (2020).

## 4 Discussion

The aim of this paper is to develop a method to assess the accuracy and utility of decisions that tinnitus patients make in their patient journey by combining concepts from SDT with HBM. In the context of assessment of diagnostic decisions, SDT has been applied successfully by using ROC-curve analysis and AUC as useful concepts that reflect the accuracy of such decisions (Swets et al., 2000). Predictive variables can be used to create statistical prediction rules with statistical methods like logistic regression. With the concepts of benefits and costs associated with decision outcomes, an optimal decision criterion or threshold can be found that optimizes the utility of diagnostic decisions. We have applied the same concepts to decisions that tinnitus patients make when freely deciding between audiological care and psychosocial counseling as complementary treatment options. HBM connects the predictive variables from SDT to the concept of perceived severity as driver of health care decisions. Also, the concepts of benefits and costs associated with decision outcomes from SDT are connected to perceived benefits and costs of health care interventions that patients may use in deliberation about health care decisions. We are aware that more elaborate models than HBM have been developed that may also account for interaction with a patients social network. For tinnitus patients, however, perceived severity can be measured by a single questionnaire that measures tinnitus impact. This is a pragmatic motivation to use HBM with hearing loss and baseline THI-score as a first order approximation. THI is a patient related outcome measure that measures the subjective impact of tinnitus on daily live. Hearing loss was selected as potential driver because there is a strong association with tinnitus help seeking (Rademaker et al., 2021b), hearing aid uptake (Vestergaard Knudsen et al., 2010), and treating hearing loss may also have a positive impact on tinnitus (van Heteren et al., 2021).

Patient decisions about treatments cannot be studied with randomized controlled trials, as participants are randomly assigned to the different treatment groups. The aim is to compare groups that only differ in treatment but are alike for all other variables. Although this removes selection bias, the downside is that patient decisions are also removed. Therefore, assessment of the quality of patient decisions in SDM can only be performed on data collected in clinical practice. This will inevitably introduce group differences, as is demonstrated in Table 1. Differences in hearing loss and baseline THI-score are essential as they are the predictive variables of the model. Differences between groups in age, baseline GHSI-score, type of hearing loss, referring medical specialty and non-hearing related problems are possible confounders, as is the length of the evaluation period. In part 2 we will analyze the effect of age, sex and side of tinnitus perception (left ear, right ear or central) on the quality of decisions. In future studies, the model can also be expanded to include any predictive variable that will increase the performance of the statistical prediction rule. Our results showed that the groups were alike for all the tinnitus related variables. Only the group with no SEDEP & PCU seemed to have more participants with tinnitus duration of 2–6 months than the other groups, although statistical significance was not reached.

The ANOVAs of the primary outcome measure, change and relative change in THI-score, showed no treatment effect. Group means for only SEDEP and only PCU were comparable to treatment effects found in a randomized controlled trial performed in the Netherland were specialized treatment based on CBT was compared with usual care (Cima et al., 2012). The usual care provided, was similar to the audiological care that was provided in this study. We included only 143 participants in our study, while the randomized controlled trial compared 247 patients that received usual care to 245 patients that were assigned to specialized care. Therefore, a lack of power in our study may explain why we found no treatment effect.

Logistic regression with Bayesian inference showed that hearing loss is a driver for SEDEP and baseline THI-score is a driver for PCU. The ROC-curves constructed with fitted and logit transformed cumulative distribution functions showed a very different trajectory for baseline THI-score and PCU compared to an example based on an equal variance SDT. This is the result of a considerable difference in value of the slope parameters for populations *S*_1_ and *S*_2_. For hearing loss and SEDEP, after imputation, the trajectory was more like the equal variance model. The accuracy of both decisions is small, although decisions about PCU are more accurate than decisions about SEDEP. This is in line with findings in literature indicating that there is some evidence that CBT is more effective than audiological care (Fuller et al., 2020), and there is no evidence for the effectiveness of sound enrichment (Sereda et al., 2018). The negative value of the accuracy parameter 2 *μ*/s for PCU indicates that lower baseline THI-scores are associated with a higher probability of experiencing a clinically important change after treatment. This is the result of the difference in slope value for *S*_1_ and *S*_2_. As can be seen from Equation S.2.3, the slope parameter also affects the location of the mode of the cumulative probability distribution. Apparently the mode of *S*_1_ is shifted towards higher baseline THI-scores and the mode of *S*_2_ towards lower scores.

Apart from accuracy, decision criterion is another aspect of decision performance (Swets et al., 2000). For psychosocial counseling however, the decision criterion is outside the range of baseline THI-scores (either below 0 points or above 100 points) based on the data set with imputation. This indicates that the probability of benefitting from psychosocial counseling is always larger than 1, which is in line with the evidence in favor of CBT found in the literature. For audiological care we found an unbiased decision criterion or decision threshold of 37 dB hearing loss with imputation. For complete case analysis we found a decision threshold of 89 points for PCU and 93 dB for SEDEP. Imputation did not change median THI-scores of the treatment groups significantly, except for the post-treatment score of the group that chose no intervention. As we only used true positive and false positive cases in our SDT model, thus all treatment groups except the group without intervention, this has not affected our analysis.

A first limitation is the number of missing responses for the questionnaires. We therefore had to impute missing values or only perform a complete case analysis. Imputation had no effect on group medians of baseline THI-score. It had only a small effect on group medians of post-treatment THI-score, and group means of change in THI-score which is our primary outcome measure. It did have a significant effect on GHSI-score, but after imputation the results are more in line, however, with what can be expected based on the HBM. Generally, imputation removes bias due to the dependence of the probability of missing data on participant characteristics. Single imputation, as we have performed, removes this bias but leads to an underestimation of standard errors or *p*-values and thus in an overestimation of the precision of the study (Donders et al., 2006). This overestimation can be reduced by multiple imputation. The goal of our study, however, is model development and all statistical analyses are exploratory. They were performed merely to assess whether group differences exist in mean values of the variables. Increasing *p*-values by multiple imputation would therefore not change results in a fundamental way. With multiple imputation, some observed group differences in mean values of variables may turn out to be statistically insignificant. Single imputation may have resulted in an underestimation BCIs of the estimated model parameters with Bayesian inference. However, imputation has not changed the results of the Bayesian inference in a fundamental way. Multiple imputation may result in less variance of the posterior distribution of parameter values. It is unlikely to affect the MLEs, however.

Another limitation of our study is that perceived benefits and costs have not been quantified. We can therefore not separate base rate of treatment success from perceived barriers and costs (see Equation 1). Only randomized controlled trials can produce unbiased estimates of these base rates. Together with a validated questionnaire mapping perceived benefits and costs, such an approach may provide valuable information about *a priori* treatment efficacy and perceived benefits and cost. This can assist clinicians to support patients in SDM and may help to better match treatment options to individual patient needs.

Also, the recruitment of participants in an outpatient audiology clinic has led to selection bias as 97% of the participant had hearing impairment to some degree, and thus may have benefited from treating hearing loss. This may have resulted in an overestimation of the efficacy of TST, and thus may compromise the generalizability of our findings. As a result, decision thresholds found in this study should be interpreted with care, as further research is needed to validate our findings in different patient groups.

To improve SDM, Stiggelbout et al. (2012) suggests several questions to support deliberation. Two questions are especially of interest for probing the decision criterion: how do the benefits of different treatment options compare? And how do the costs compare? Based on the ratios that determine the decision criterion in SDT (Equation 1) and translating them to the concepts of HBM, these questions can be made more specific for probing the decision criterion:

If treatment option X turns out to be successful:

- What are the perceived benefits associated with choosing treatment option X?
- What are the perceived costs associated with not choosing treatment option X?

If treatment option X turns out to be unsuccessful:

- What are the perceived benefits associated with not choosing treatment option X?
- What are the perceived costs associated with choosing treatment option X?

Quantifying the answers to these questions, for example on a visual analogue scale, allows calculation of the above-mentioned ratio of costs and benefits associated with TP, FP, TN, and FN decisions.

The question remains how to define success. The clinician and patient may have very different definitions of success, as they may use very different drivers for health-related decisions. Supporting deliberation in SDM may thus involve a discussion where the clinician specifies his/her definition of success, how probable it is that treatment option X will be successful, and what the benefits and costs are based on clinical experience. The patient could do the same with respect to his/her definition of success (ideally associated with validated patient related outcome measures) and driver(s) of decisions but should additionally be probed for expected benefits and costs associated with the treatment options, preferably with validated questionnaires. Use or development of appropriate and validated patient related outcome measures and questionnaires that probe benefits and costs of treatments may thus be crucial for high quality SDM, especially in case of professional equipoise.

## Data Availability

The raw data supporting the conclusions of this article will be made available by the authors, without undue reservation.

## References

[ref1] Armstrong R. A. (2014). When to use the Bonferroni correction. Ophthal. Physiol. Optics 34, 502–508. doi: 10.1111/opo.12131 24697967

[ref2] Baguley D. McFerran D. Hall D. (2013). Tinnitus. Lancet 382, 1600–1607. doi: 10.1016/S0140-6736(13)60142-7 23827090

[ref3] Bankstahl U. S. Elkin E. P. Gebauer A. Görtelmeyer R. (2012). Validation of the THI-12 questionnaire for international use in assessing tinnitus: a multi-Centre, prospective, observational study. Int. J. Audiol. 51, 671–677. doi: 10.3109/14992027.2011.653448, PMID: 22339398

[ref4] Cima R. F. F. Andersson G. Schmidt C. J. Henry J. A. (2014). Cognitive-behavioral treatments for tinnitus: a review of the literature. J. Am. Acad. Audiol. 25, 029–061. doi: 10.3766/jaaa.25.1.4 24622860

[ref5] Cima R. F. Maes I. H. Joore M. A. Scheyen D. J. El Refaie A. Baguley D. M.. (2012). Specialised treatment based on cognitive behaviour therapy versus usual care for tinnitus: a randomised controlled trial. 379. Available at: www.thelancet.com 10.1016/S0140-6736(12)60469-3 22633033

[ref6] Cima R. F. F. Mazurek B. Haider H. Kikidis D. Lapira A. Noreña A.. (2019). A multidisciplinary European guideline for tinnitus: diagnostics, assessment, and treatment. HNO 67, 10–42. doi: 10.1007/s00106-019-0633-7, PMID: 30847513

[ref7] De Ridder D. Vanneste S. Weisz N. Londero A. Schlee W. Elgoyhen A. B.. (2014). An integrative model of auditory phantom perception: tinnitus as a unified percept of interacting separable subnetworks. Neurosci. Biobehav. Rev. 44, 16–32. doi: 10.1016/j.neubiorev.2013.03.021, PMID: 23597755

[ref8] DeCarlo L. T. (1998). Signal detection theory and generalized linear models. Psychol. Methods. 3, 186–205.

[ref9] DeCarlo L. T. (2010). On the statistical and theoretical basis of signal detection theory and extensions: unequal variance, random coefficient, and mixture models. J. Math. Psychol. 54, 304–313. doi: 10.1016/j.jmp.2010.01.001

[ref10] Donders A. R. T. van der Heijden G. J. M. G. Stijnen T. Moons K. G. M. (2006). Review: a gentle introduction to imputation of missing values. J. Clin. Epidemiol. 59, 1087–1091. doi: 10.1016/j.jclinepi.2006.01.014, PMID: 16980149

[ref11] Elwyn G. J. Edwards A. Kinnersley P. Grol R. (2000). Shared decision making and the concept of equipoise: the competences of involving patients in healthcare choices. Br. J. Gen. Pract. 50, 892–897. 11141876 PMC1313854

[ref12] Elwyn G. Frosch D. Thomson R. Joseph-Williams N. Lloyd A. Kinnersley P.. (2012). Shared decision making: a model for clinical practice. J. Gen. Intern. Med. 27, 1361–1367. doi: 10.1007/s11606-012-2077-6, PMID: 22618581 PMC3445676

[ref13] Elwyn G. Laitner S. Coulter A. Walker E. Watson P. Thomson R. (2010). Implementing shared decision making in the NHS. BMJ 341, c5146–c5972. doi: 10.1136/bmj.c5146 20947577

[ref14] Fuller T. Cima R. Langguth B. Mazurek B. Vlaeyen J. W. S. Hoare D. J. (2020). Cognitive behavioural therapy for tinnitus. Cochrane Database Syst. Rev. 2020:CD012614. doi: 10.1002/14651858.CD012614.pub2, PMID: 31912887 PMC6956618

[ref15] Galazyuk A. V. Wenstrup J. J. Hamid M. A. (2012). Tinnitus and underlying brain mechanisms. Curr. Opin. Otolaryngol. Head Neck Surg. 20, 409–415. doi: 10.1097/MOO.0b013e3283577b81, PMID: 22931904 PMC3886369

[ref16] Janz N. K. Becker M. H. (1984). The health belief model: a decade later. Health Educ. Q. 11, 1–47. doi: 10.1177/109019818401100101, PMID: 6392204

[ref17] Jarach C. M. Lugo A. Scala M. Van Den Brandt P. A. Cederroth C. R. Odone A.. (2022). Global prevalence and incidence of tinnitus a systematic review and Meta-analysis supplemental content. JAMA Neurol. 79, 888–900. doi: 10.1001/jamaneurol.2022.2189, PMID: 35939312 PMC9361184

[ref18] Knipper M. van Dijk P. Schulze H. Mazurek B. Krauss P. Scheper V.. (2020). The neural bases of tinnitus: lessons from deafness and cochlear implants. J. Neurosci. 40, 7190–7202. doi: 10.1523/JNEUROSCI.1314-19.2020 32938634 PMC7534911

[ref19] Krauss P. Tziridis K. Metzner C. Schilling A. Hoppe U. Schulze H. (2016). Stochastic resonance controlled upregulation of internal noise after hearing loss as a putative cause of tinnitus-related neuronal hyperactivity. Front. Neurosci. 10. doi: 10.3389/FNINS.2016.00597/ABSTRACT PMC5187388 28082861

[ref20] Langguth B. Kreuzer P. M. Kleinjung T. De Ridder D. (2013). Tinnitus: causes and clinical management. Lancet Neurol. 12, 920–930. doi: 10.1016/S1474-4422(13)70160-1 23948178

[ref21] Macmillan N. A. Creelman C. D. (1990). Response Bias: characteristics of detection theory, threshold theory, and “nonparametric” indexes. Psychol. Bull. 107, 401–413. doi: 10.1037/0033-2909.107.3.401

[ref22] Macmillan N. A. Creelman C. D. (2004). Detection and discrimination of compound stimuli: tools for multidimensional detection theory. In Detect. Theor. 3rd ed. Psychology Press. eBook.

[ref23] Marks E. Smith P. McKenna L. (2019). Living with tinnitus and the health care journey: an interpretative phenomenological analysis. Br. J. Health Psychol. 24, 250–264. doi: 10.1111/bjhp.12351, PMID: 30609202

[ref24] Newman C. W. Jacobson G. P. Spitzer J. B. (1996). Development of the tinnitus handicap inventory. Arch. Otolaryngol. 122, 143–148. doi: 10.1001/archotol.1996.01890140029007 8630207

[ref25] Rademaker M. M. Essers B. A. B. Stokroos R. J. Smit A. L. Stegeman I. (2021a). What tinnitus therapy outcome measures are important for patients?– a discrete choice experiment. Front. Neurol. 12, 1–10. doi: 10.3389/fneur.2021.668880, PMID: 34113313 PMC8185356

[ref26] Rademaker M. M. Stegeman I. Brabers A. E. M. de Jong J. D. Stokroos R. J. Smit A. L. (2021b). Differences in characteristics between people with tinnitus that seek help and that do not. Sci. Rep. 11:22949. doi: 10.1038/s41598-021-01632-5, PMID: 34824285 PMC8616930

[ref27] Schilling A. Sedley W. Gerum R. Metzner C. Tziridis K. Maier A.. (2023). Predictive coding and stochastic resonance as fundamental principles of auditory phantom perception. Brain 146, 4809–4825. doi: 10.1093/brain/awad255, PMID: 37503725 PMC10690027

[ref28] Searchfield G. D. (2021). Sense and sensibility: a review of the behavioral neuroscience of tinnitus sound therapy and a new typology. Curr. Top. Behav. Neurosci. 51, 213–247. doi: 10.1007/7854_2020_183 33547596

[ref29] Sedley W. Friston K. J. Gander P. E. Kumar S. Griffiths T. D. (2016). An integrative tinnitus model based on sensory precision the symptomatology and pathophysiology of tinnitus. Trends Neurosci. 39, 799–812. doi: 10.1016/j.tins.2016.10.004, PMID: 27871729 PMC5152595

[ref30] Sereda M. Xia J. El Refaie A. Hall D. A. Hoare D. J. (2018). Sound therapy (using amplification devices and/or sound generators) for tinnitus. Cochrane Database Syst. Rev. 2018:CD013094. doi: 10.1002/14651858.CD013094.pub2, PMID: 30589445 PMC6517157

[ref31] Stiggelbout A. M. Van Der Weijden T. De Wit M. P. T. Frosch D. Légaré F. Montori V. M.. (2012). Shared decision making: really putting patients at the Centre of healthcare. BMJ 344, 1–6. doi: 10.1136/bmj.e256, PMID: 22286508

[ref32] Swets J. A. Dawes R. M. Monahan J. (2000). Psychological science can improve diagnostic decisions. Psychol. Sci. Public Interest 1, 1–26. doi: 10.1111/1529-1006.001 26151979

[ref34] van Heteren J. A. A. Arts R. A. G. J. Killian M. J. P. Assouly K. K. S. van de Wauw C. Stokroos R. J.. (2021). Sound therapy for cochlear implant users with tinnitus. Int. J. Audiol. 60, 374–384. doi: 10.1080/14992027.2020.1832266 33074733

[ref35] Vestergaard Knudsen L. Öberg M. Nielsen C. Naylor G. Kramer S. E. (2010). Factors influencing help seeking, hearing aid uptake, hearing aid use and satisfaction with hearing aids: a review of the literature. Trends Amplif. 14, 127–154. doi: 10.1177/1084713810385712, PMID: 21109549 PMC4111466

[ref36] Zeman F. Koller M. Figueiredo R. Aazevedo A. Rates M. Coelho C.. (2011). Tinnitus handicap inventory for evaluating treatment effects: which changes are clinically relevant? Otolaryngol. Head Neck Surg. 145, 282–287. doi: 10.1177/0194599811403882 21493265

