# Supplementary material for: Combining unequal variance signal detection theory with the health belief model to optimize shared decision making in tinnitus patients: part 1—model development
Source: Front Neurosci. 2024 Dec 4;18:1451741. doi: 10.3389/fnins.2024.1451741 (PMC11653419; doi:10.3389/fnins.2024.1451741)
Supplement: Supplementary file 1 [file Data_Sheet_1.docx]

**Supplement 1 – Analysis of Compound Decisions**

Participants did not make a single decision that determines treatment outcome. They made a compound decision regarding starting an evaluation period with a sound enrichment device and/or psychosocial counseling uptake. Following Macmillan & Creelman (2004), compound decisions require extension of the one-dimensional model to a two-dimensional model with joint probability distributions. If the decisions are made independently^[[1]](#footnote-1)^, the value of the two-dimensional probability distribution *f*(*x*,*y*) at any combination of the two axis values (*x*,*y*) is the product of the two independent distributions *f*(*x*) and *f*(*y*). Figure S.1 shows an example of a two-dimensional decision space corresponding to a compound decision assuming logistic distributions for both decisions with arbitrary axis values. Appreciating that the sum of the probabilities of a *yes* response and *no* response for decision 2 for regions I and IV equals unity, it is easily demonstrated that summing the joint probabilities for these regions yields the probability for decision 1, e.g.

*P_y_*_,_*_y_* + *P_y_*_,_*_n_* = *P*(decision 1 = yes | decision 2 = yes) + *P*(decision 1 = yes | decision 2 = no) = …

*P*(decision 1 = yes)∙*P*(decision 2 = yes) + *P*(decision 1 = yes)∙*P*(decision 2 = no) = …

*P*(decision 1 = yes)∙{ *P*(decision 2 = yes) + *P* (decision 2 = no) } = …

*P*(decision 1 = yes) ∙1 =  *P*(decision 1 = yes)

Likewise, summing the joint probabilities for regions I and II yields the probability for decision 2. This can be done separately for true positive cases and false positive cases resulting in the following set of equations:

*FP*_1_ = *FP_y_*_,_*_y_* + *FP_y_*_,_*_n_* = *n*_1,_*_y_*/*n* (S.1.1a)

*TP*_1_ = *TP_y_*_,_*_y_* + *TP_y_*_,_*_n_* = *m*_1,_*_y_*/*m* (S.1.2b)

*FP*_2_ = *FP_y_*_,_*_y_* + *FP_n_*_,_*_y_* = *n*_2,_*_y_*/*n* (S.1.3c)

*TP*_2_ = *TP_y_*_,_*_y_* + *TP_n_*_,_*_y_* = *m*_2,_*_y_*/*m* (S.1.4d)

Where *FP*_i_ is the probability of a false positive response for decision *i*, *TP*_i_ is the probability of a true positive response for decision *i*, the subscripts *y*,*y* indicate a *yes* response for both decisions, subscripts *y*,*n* indicate a *yes* response for decision 1 and a *no* response for decision 2 and mutatis mutandis for subscripts *n*,*y* and *n*,*n*. Furthermore, *n_i,y_* is the number of false positive cases for decision *i* and *n* is the total number of participants belonging to population *S*_1_. Likewise, *m_i_*_,_*_y_* is the number of true positive cases for decision *i* and *m* is the total number of participants belonging to S_2_.

**Figure caption**

Figure S.1.1 Top: compound probability density functions corresponding to *no clinically important change* (*S*_1_) and *clinically important change* (*S*_2_) in a patient related outcome measure. Decision axes 1 and 2 represent for decisions 1 and 2 respectively, the driver magnitude expressed in arbitrary units. Bottom: projection of the probability contours on the *z* = 0 plane. Also projected are the locations of the modes of *S*_1_ and *S*_2_ with coordinates (-*µ*_1_, - *µ* _2_) and (+*µ* _1_, + *µ* _2_), respectively. The dashed lines represent the location of unbiased decision criteria *c*_1_ and *c*_2_ for decisions 1 and 2. Four different regions can be identified that are delimited by the decision criteria *c*_1_ and *c*_2_, see text for an explanation.

**References**

Macmillan, N. A., & Creelman, C. D. (2004). Detection and Discrimination of Compound Stimuli: Tools for Multidimensional Detection Theory. In *Detection Theory* (3rd ed., Issue 2004, pp. 146–166). Psychology Press. https://doi.org/10.4324/9781410611147-14

1. If the decisions are not made independently, it is always possible to rotate decision space such

   that two orthogonal decision axes can be constructed. [↑](#footnote-ref-1)
