# Supplementary material for: Combining unequal variance signal detection theory with the health belief model to optimize shared decision making in tinnitus patients: part 1—model development
Source: Front Neurosci. 2024 Dec 4;18:1451741. doi: 10.3389/fnins.2024.1451741 (PMC11653419; doi:10.3389/fnins.2024.1451741)
Supplement: Supplementary file 2 [file Data_Sheet_2.docx]

**Supplement 2 – Statistical Model**

Following DeCarlo (2010), the statistical model is mathematically represented as follows. The probability *P* of a false positive (FP) response, for decision *i* = {audiological care, psychosocial counseling} and driver *j* = {hearing loss, baseline THI-score}, is the probability *p* of a yes-response conditional on belonging to population *S*_1_ (ΔTHI-score > -7 points; see Figure 2) and decision criterion *c_i_*_,_*_j_* that is associated to the utility of the decision process, and is given by,

$P_{i,j}^{FP}= p_{i,j}(Y_{i,j}|S_{1},c_{i,j})=\int_{c_{i,j}}^{\infty} f_{i,j}^{S1}\left( x_{j} \right)dx_{j}=1-F_{i,j}^{S1}(c_{i,j})$, (S.2.1a)

Where *Y_i_*_,_*_j_* is a yes-response for decision *i* and driver *j, x_j_* is the magnitude of driver *j*, *f_i_*_,_*_j_*(*x_j_*) is the probability density function of *S*_1_ and *F_i_*_,_*_j_*(*x_j_*) is the corresponding cumulative distribution function. Likewise, the probability of a true positive (TP) response, is the probability of a yes-response conditional on belonging to population *S*_2_ (ΔTHI-score < -7 points; see Figure 2) and decision criterion *c_i_*_,_*_j_*,

$P_{i,j}^{TP}= p_{i,j}(Y_{i,j}|S_{2},c_{i,j})=\int_{c_{i,j}}^{\infty} f_{i,j}^{S2}\left( x_{j} \right)dx_{j}=1-F_{i,j}^{S2}(c_{i,j})$, (S.2.1b)

Based on Choice Theory, e.g. Macmillan & Creelman (1990) and considering that *Y_i_*_,_*_j_* is binomial, we assumed underlying logistic distributions. Hence, *f_i_*_,_*_j_*(*x_i_*) is given by,

$f_{i,j}(x_{j};\mu_{i,j},s_{i,j})={e^{-(x_{j}-\mu_{i,j})/s_{i,j}}}/\left\{ s_{i,j}\left( 1+e^{-(x_{j}-\mu_{i,j})/s_{i,j}} \right)^{2} \right\}$, (S.2.2)

and *F_i_*_,_*_j_*(*x_i_*) by

$F_{i,j}(x_{j};\mu_{i,j},s_{i,j})=1/\left\{ 1+e^{-(x_{j}-\mu_{i,j})/s_{i,j}} \right\}$, (S.2.3)

where *μ_i_*_,_*_j_* is the location parameter of the distribution and *s_i_*_,_*_j_* the scale parameter, which is proportional to the variance of the distribution, i.e. variance = (*s*π)^2^/3. Inserting Equation (S.2.3) into Equations (S.2.1a) and (S.2.1b) yields, after elementary algebraic manipulation, the following relation for the probabilities of a FP or TP response and the respective parameters of the distribution functions,

$s_{i,j}^{S1}\cdot ln\left( {P_{i,j}^{FP}}/\left\{ 1-P_{i,j}^{FP} \right\} \right)= -c_{i,j}+\mu_{i,j}^{S1}$, (S.2.4a)

$s_{i,j}^{S2}\cdot ln\left( {P_{i,j}^{TP}}/\left\{ 1-P_{i,j}^{TP} \right\} \right)= -c_{i,j}+\mu_{i,j}^{S2}$. (S.2.4b)

Where *ln* is the natural logarithm. Now subtracting Equations (S.2.4a) and (S.2.4b) gives, again after some elementary algebraic manipulation,

$logit\left( P_{i,j}^{TP} \right)= {s_{i,j}^{S1}}/{s_{i,j}^{S2}}\cdot logit\left( P_{i,j}^{FP} \right)+{2\mu_{i,j}}/{s_{i,j}^{S2}}$, (S.2.5a)

${2\mu}_{i,j}=\mu_{i,j}^{S2}- \mu_{i,j}^{S1}$, (S.2.5b)

where, $logit\left( z \right)= ln\left( z/\left\{ 1-z \right\} \right)$ is the link function and 2*µ_i_*_,_*_j_* is the distance between the modes of *S*_1_ and *S*_2_, and portrays the accuracy of the decision process. As Decarlo (1998) has shown, signal detection models with different underlying distribution functions can be obtained by using different link functions. For an equal variance signal detection model, it will hold that $s_{i,j}^{S_{1}}=s_{i,j}^{S_{2}}$, while for an unequal variance signal detection model $s_{i,j}^{S_{1}}\neq s_{i,j}^{S_{2}}$.

**References**

Decarlo, L. T. (1998). Psychological Methods Signal Detection Theory and Generalized Linear Models. *Psychological Methods*, *3*(2), 186–205.

DeCarlo, L. T. (2010). On the statistical and theoretical basis of signal detection theory and extensions: Unequal variance, random coefficient, and mixture models. *Journal of Mathematical Psychology*, *54*(3), 304–313. https://doi.org/10.1016/j.jmp.2010.01.001

Macmillan, N. A., & Creelman, C. D. (1990). Response Bias: Characteristics of Detection Theory, Threshold Theory, and “Nonparametric” Indexes. *Psychological Bulletin*, *107*(3), 401–413. https://doi.org/10.1037/0033-2909.107.3.401
