# Supplementary material for: Combining unequal variance signal detection theory with the health belief model to optimize shared decision making in tinnitus patients: part 1—model development
Source: Front Neurosci. 2024 Dec 4;18:1451741. doi: 10.3389/fnins.2024.1451741 (PMC11653419; doi:10.3389/fnins.2024.1451741)
Supplement: Supplementary file 3 [file Data_Sheet_3.docx]

**Supplement 3 – Bayesian Inference**

According to Bayes’ theorem, e.g. Box & Tiao (1992), the joint posterior probability distribution of parameters ***θ*** = (*θ*_1_,*θ*_2_) given observations ***y*** = (*y*_1_, … , *y_k_*) is given by

$p\left( \boldsymbol{\theta} \right|\boldsymbol{y})=K^{-1}l(\boldsymbol{\theta|y)}p(\boldsymbol{\theta)}$ (S.3.1a)

$K= \iint l(\boldsymbol{\theta|y)}d\boldsymbol{\theta}$ (S.3.1b)

The probability of parameter values ***θ*** occurring as function of the probability *π* of a *yes* response (y = 1) is given by the Bernoulli distribution,

$p\left( \boldsymbol{\theta} \right|\pi)= \pi^{y}{(1-\pi)}^{1-y}$, (S.3.2)

where y = 0 for a *no* response. The joint posterior probability distributions for *S*_1_ and *S*_2_ for all observations for a given decision *i* and stimulus *j* are therefore given by,

$p_{S1}\left( \boldsymbol{\theta}_{S1} | \boldsymbol{\pi}_{\boldsymbol{S}\boldsymbol{1}} \right)\boldsymbol{=}K^{-1}\prod_{\kappa=1}^{k} {(\pi_{\kappa}^{S1})}^{y}{(1-\pi_{\kappa}^{S1})}^{1-y}$ (S.3.3a)

With

$\pi_{\kappa}^{S1}=1/\left\{ 1+e^{-\left( x_{k}\cdot\theta_{2}^{S_{1}}-\theta_{1}^{S_{1}} \right)} \right\}$ (S.3.3b)

and

$p_{S2}\left( \boldsymbol{\theta}_{S2} | \boldsymbol{\pi}_{S2} \right)\boldsymbol{=}K^{-1}\prod_{\kappa=1}^{k} {(\pi_{\kappa}^{S2})}^{y}{(1-\pi_{\kappa}^{S2})}^{y}$ (S.3.3c)

$\pi_{\kappa}^{S2}=1/\left\{ 1+e^{-\left( x_{k}\cdot\theta_{2}^{S_{2}}-\theta_{1}^{S_{2}} \right)} \right\}$ (S.3.3d)

Where *θ*_1_ ∈ (-∞,∞) is labeled *intercept* and *θ*_2_ ∈ (0,∞) is labeled *slope*. Inserting the data with and without imputation in Equations S.3.3a-d yields estimates of the parameters. Figures S.3.1 and S.3.2 show the graphical results. The numerical values are presented in Table S.3.1 (imputation) and Table S.3.2 (complete case). The maximum likelihood estimate (MLE) of a set of parameters is determined by taking the coordinates of the mode of the posterior joint probability distribution (see top panels in Figures S.3.1 and S.3.2). The variance (Var) of a parameter, say slope (see middle panels of Figures S.3.1 and S.3.2), is determined by calculating the variance of the marginal posterior probability distributions with the other parameter, in this case intercept, held constant at its MLE (i.e. along projections a and b). Vice versa for the intercept parameter (see bottom panels of Figures S.3.1 and S.3.2), i.e. along projections c and d. The lower bound and upper bound of a parameter are given by MLE ± 2*sqrt(Var). For each parameter the Bayesian Confidence Interval (BCI) gives the probability of that parameter having a value between its lower and upper bound, conditionally on the other parameter having a value equal to its MLE.

The effect of imputation is most clear from Figures S.3.1 and S.3.2. The left panel in the middle row of Figure S.3.1 shows that imputation affects estimation of the slope parameter for SEDEP and hearing loss most. Both the MLE and variance are affected. After imputation the MLEs of the slopes of *S*_1_ and *S*_2_ are almost equal, and the variance of the posterior distribution has decreased compared to the result for complete case analysis. This decreased variance of the posterior probability distribution is seen for all parameters when results with imputation are compared to results for complete case analysis.

**References**

Box, G. E. P., & Tiao, G. C. (1992). *Bayesian Inference in Statistical Analysis* (Wiley Classical). John Wiley and Sons, Inc.

**Figures captions**

Figure S.3.1. Results of the Bayesian fitting procedure for decisions to start an evaluation period (SEDEP) and psychosocial counseling (PCU) with hearing loss as driver. Top panels show contour plots of the joint posterior probability density functions of the parameters intercept (ordinate) and slope (abscissa) of populations *S*_1_ (no clinically important change, solid lines) and *S*_2_ (clinically important change, dashed lines) with imputation. Circles mark the modes of the distributions and correspond to the maximum likelihood estimates of intercept and slope. The line marked *a* indicates the projection of the marginal distribution of the slope parameter for *S*_1_, conditional on the intercept for *S*_1_ having a value equal to its maximum likelihood estimate. Likewise, line *b* marks the same projection for *S*_2_.

The projections along lines *a* and *b* of the marginal probability density functions for the slope parameters for *S*_1_ and *S*_2_ are shown in the middle row panels. Solid lines mark results with imputation and dashed lines for complete case analysis. The vertical dashed line located at slope = 0 indicates the lower bound of the integration range to calculate the probability of slope > 0, which is used for statistically testing the rejection of the null hypothesis.

Lines *c* and *d* in the top panels mark the projections of the marginal probability density functions of the intercept, conditional on the slope having a value equal to its maximum likelihood estimate. The projections along line *c* and *d* of the marginal probability density functions for the intercept parameters for *S*_1_ and *S*_2_ are shown in the bottom row panels. Again, solid lines mark results with imputation and dashed lines for complete case analysis.

Figure S.3.2. Results of the Bayesian fitting procedure for decisions to start an evaluation period (SEDEP) and psychosocial counseling (PCU) with baseline THI-score as driver. Top panels show contour plots of the joint posterior probability density functions of the parameters intercept (ordinate) and slope (abscissa) of populations *S*_1_ (no clinically important change, solid lines) and *S*_2_ (clinically important change, dashed lines) with imputation. Circles mark the modes of the distributions and correspond to the maximum likelihood estimates of intercept and slope. The line marked *a* indicates the projection of the marginal distribution of the slope parameter for *S*_1_, conditional on the intercept for *S*_1_ having a value equal to its maximum likelihood estimate. Likewise, line *b* marks the same projection for *S*_2_.

The projections along lines *a* and *b* of the marginal probability density functions for the slope parameters for *S*_1_ and *S*_2_ are shown in the middle row panels. Solid lines mark results with imputation and dashed lines for complete case analysis. The vertical dashed line located at slope = 0 indicates the lower bound of the integration range to calculate the probability of slope > 0, which is used for statistically testing the rejection of the null hypothesis.

Lines *c* and *d* in the top panels mark the projections of the marginal probability density functions of the intercept, conditional on the slope having a value equal to its maximum likelihood estimate. The projections along line *c* and *d* of the marginal probability density functions for the intercept parameters for *S*_1_ and *S*_2_ are shown in the bottom row panels. Again, solid lines mark results with imputation and dashed lines for complete case analysis.

Table S.3.1. Numerical values of the parameters intercept and slope for populations *S*_1_ (no clinically significant improvement) and *S*_2_ (clinically significant improvement) for drivers hearing level and baseline THI-score with imputation. MLE is the maximum likelihood estimate, Var is the variance of the distribution of the parameter, the lower and upper bounds of a parameter are defined as MLE ± 2*sqrt(Var). BCI is the Bayesian Confidence Interval which is the probability of a parameter having a value between the upper and lower bound. The probability of slope > 0, is calculated with 0 as lower bound and ∞ as upper bound.

^*^ p < 0.01. SEDEP: starting an evaluation period; PCU: psychosocial counseling uptake.

| **driver** | **decision** | **parameter** | **# participants** | **MLE** | **Var** | **lower bound** | **upper bound** | **BCI** | **prob slope > 0** |
| --- | --- | --- | --- | --- | --- | --- | --- | --- | --- |
| **Hearing Loss** | **SEDEP** | intercept *S*_2_ | 71 | -6.130E-01 | 7,672E-02 | -1.167E+00 | -5.907E-02 | 0.951 |  |
|  |  | slope *S*_2_ | 71 | 2.722E-02 | 2.183E-05 | 1.787E-02 | 3.656E-02 | 0.947 | 1.000^*^ |
|  |  | intercept *S*_1_ | 72 | -3.906E-01 | 8.812E-02 | -9.843E-01 | 2.031E-01 | 0.949 |  |
|  |  | slope *S*_1_ | 72 | 2.966E-02 | 2.874E-05 | 1.894E-02 | 4.038E-02 | 0.946 | 1.000^*^ |
|  | **PCU** | intercept *S*_2_ | 71 | -4.167E-01 | 5.861E-02 | -9.008E-01 | 6.754E-02 | 0.940 |  |
|  |  | slope *S*_2_ | 71 | 1.598E-03 | 1.466E-05 | -6.059E-03 | 9.255E-03 | 0.950 | 0.645 |
|  |  | intercept *S*_1_ | 72 | -1.137E+00 | 6.308E-02 | -1.639E+00 | -6.343E-01 | 0.941 |  |
|  |  | slope *S*_1_ | 72 | 7.983E-03 | 1.396E-05 | 5.108E-04 | 1.546E-02 | 0.948 | 0.979 |
| **Baseline THI-score** | **SEDEP** | intercept *S*_2_ | 71 | 4.357E-01 | 7.172E-02 | -9.994E-02 | 9.713E-01 | 0.951 |  |
|  |  | slope *S*_2_ | 71 | 1.044E-02 | 2.521E-05 | 3.947E-04 | 2.048E-02 | 0.949 | 0.983 |
|  |  | intercept *S*_1_ | 72 | 8.981E-01 | 7.906E-02 | 3.358E-01 | 1.460E+00 | 0.947 |  |
|  |  | slope *S*_1_ | 72 | 4.896E-03 | 1.856E-05 | -3.721E-03 | 1.351E-02 | 0.946 | 0.864 |
|  | **PCU** | intercept *S*_2_ | 71 | -2.199E+00 | 6.627E-02 | -2.713E+00 | -1.684E+00 | 0.944 |  |
|  |  | slope *S*_2_ | 71 | 3.622E-02 | 2.056E-05 | 2.715E-02 | 4.528E-02 | 0.947 | 1.000^*^ |
|  |  | intercept *S*_1_ | 72 | -1.331E+00 | 6.417E-02 | -1.837E+00 | -8.238E-01 | 0.948 |  |
|  |  | slope *S*_1_ | 72 | 1.834E-02 | 3.459E-05 | 6.577E-03 | 3.010E-02 | 0.950 | 0.999^*^ |

Table S.3.2. Numerical values of the parameters intercept and slope for populations *S*_1_ (no clinically significant improvement) and *S*_2_ (clinically significant improvement) for drivers hearing level and baseline THI-score for complete case analysis. MLE is the maximum likelihood estimate, Var is the variance of the distribution of the parameter, the lower and upper bounds of a parameter are defined as MLE ± 2*sqrt(Var). BCI is the Bayesian Confidence Interval which is the probability of a parameter having a value between the upper and lower bound. The probability of slope > 0, is calculated with 0 as lower bound and ∞ as upper bound.

^*^ p < 0.01. SEDEP: starting an evaluation period; PCU: psychosocial counseling uptake. See appendix C for details.

| **driver** | **decision** | **parameter** | **# participants** | **MLE** | **Var** | **lower bound** | **upper bound** | **BCI** | **prob slope > 0** |
| --- | --- | --- | --- | --- | --- | --- | --- | --- | --- |
| **Hearing Loss** | **SEDEP** | intercept *S*_2_ | 47 | 3.370E-01 | 1.360E-01 | -4.005E-01 | 1.074E-00 | 0.950 |  |
|  |  | slope *S*_2_ | 47 | 1.838E-02 | 3.849E-05 | 5.969E-03 | 3.078E-02 | 0.949 | 1.000^*^ |
|  |  | intercept *S*_1_ | 44 | -7.355E-01 | 1.625E-01 | -1.542E-00 | 7.070E-02 | 0.950 |  |
|  |  | slope *S*_1_ | 44 | 3.961E-02 | 5.725E-05 | 2.448E-02 | 5.475E-02 | 0.950 | 1.000^*^ |
|  | **PCU** | intercept *S*_2_ | 47 | -2.035E-01 | 8.707E-02 | -7.936E-01 | 3.867E-01 | 0.943 |  |
|  |  | slope *S*_2_ | 47 | 2.726E-03 | 2.174E-05 | -6.599E-03 | 1.205E-02 | 0.947 | 0.707 |
|  |  | intercept *S*_1_ | 44 | -1.325E+00 | 1.037E-01 | -1.970E-00 | -6.813E-01 | 0.950 |  |
|  |  | slope *S*_1_ | 44 | 1.236E-02 | 2.209E-05 | 2.963E-03 | 2.176E-02 | 0.945 | 0.994^*^ |
| **Baseline THI-score** | **SEDEP** | intercept *S*_2_ | 47 | 1.003E-00 | 1.330E-01 | 2.740E-01 | 1.733E-00 | 0.946 |  |
|  |  | slope *S*_2_ | 47 | 7.746E-03 | 4.592E-05 | -5.807E-03 | 2.130E-02 | 0.947 | 0.872 |
|  |  | intercept *S*_1_ | 44 | 7.449E-01 | 1.358E-01 | 7.763E-03 | 1.482E+00 | 0.949 |  |
|  |  | slope *S*_1_ | 44 | 9.915E-03 | 4.035E-05 | -2.790E-03 | 2.262E-02 | 0.948 | 0.945 |
|  | **PCU** | intercept *S*_2_ | 47 | -2.038E+00 | 1.006E-01 | -2.673E+00 | -1.404E+00 | 0.947 |  |
|  |  | slope *S*_2_ | 47 | 3.853E-02 | 3.241E-05 | 2.715E-02 | 4.992E-02 | 0.947 | 1.000^*^ |
|  |  | intercept *S*_1_ | 44 | -1.794E+00 | 1.078E-01 | -2.451E+00 | -1.137E-00 | 0.946 |  |
|  |  | slope *S*_1_ | 44 | 2.736E-02 | 4.376E-05 | 1.413E-02 | 4.059E-02 | 0.951 | 1.000^*^ |
