# Supplementary figures and images for: Combining unequal variance signal detection theory with the health belief model to optimize shared decision making in tinnitus patients: part 1—model development
Source: Front Neurosci. 2024 Dec 4;18:1451741. doi: 10.3389/fnins.2024.1451741 (PMC11653419; doi:10.3389/fnins.2024.1451741)

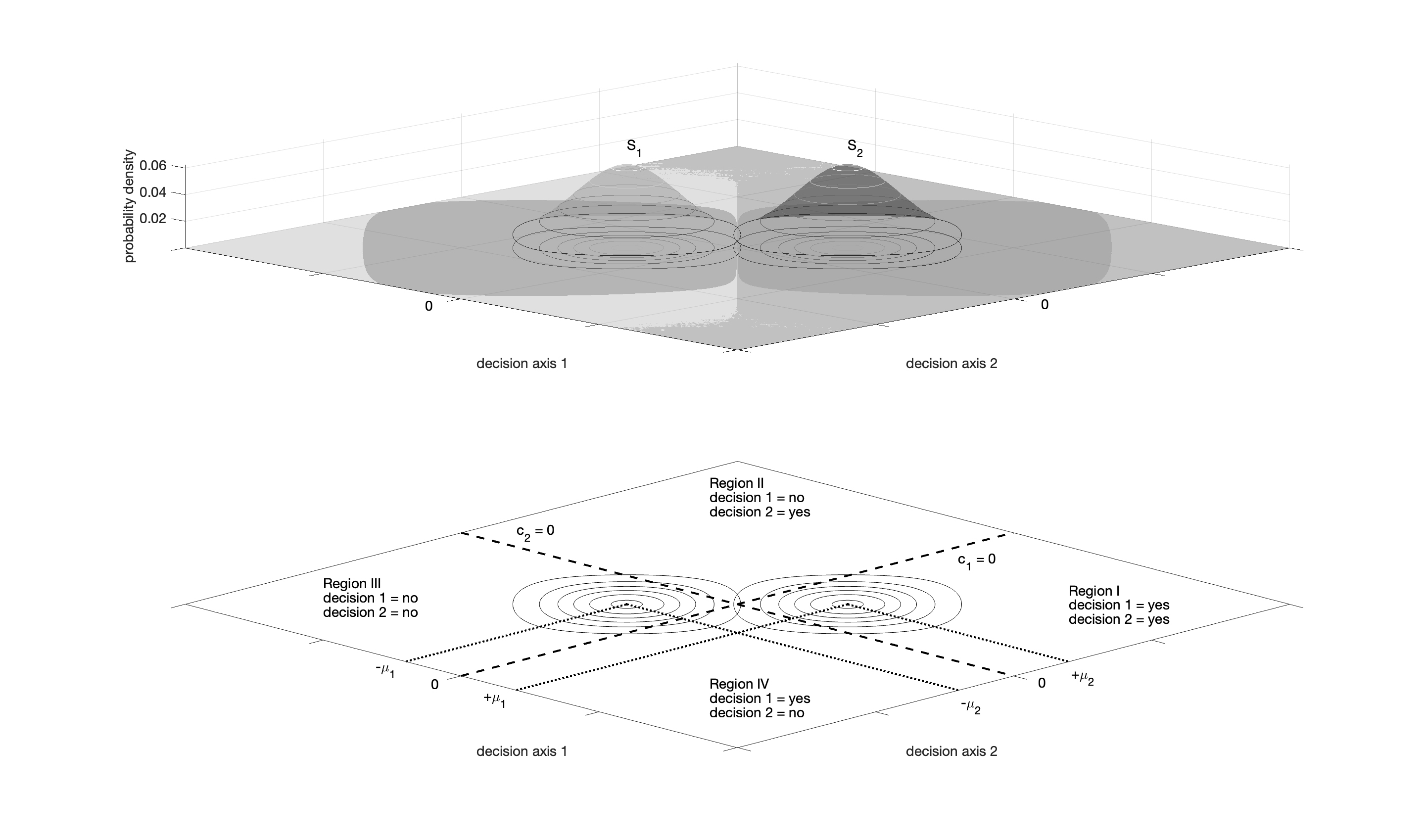

Supplement: Supplementary file 4 [file Image_1.jpg]

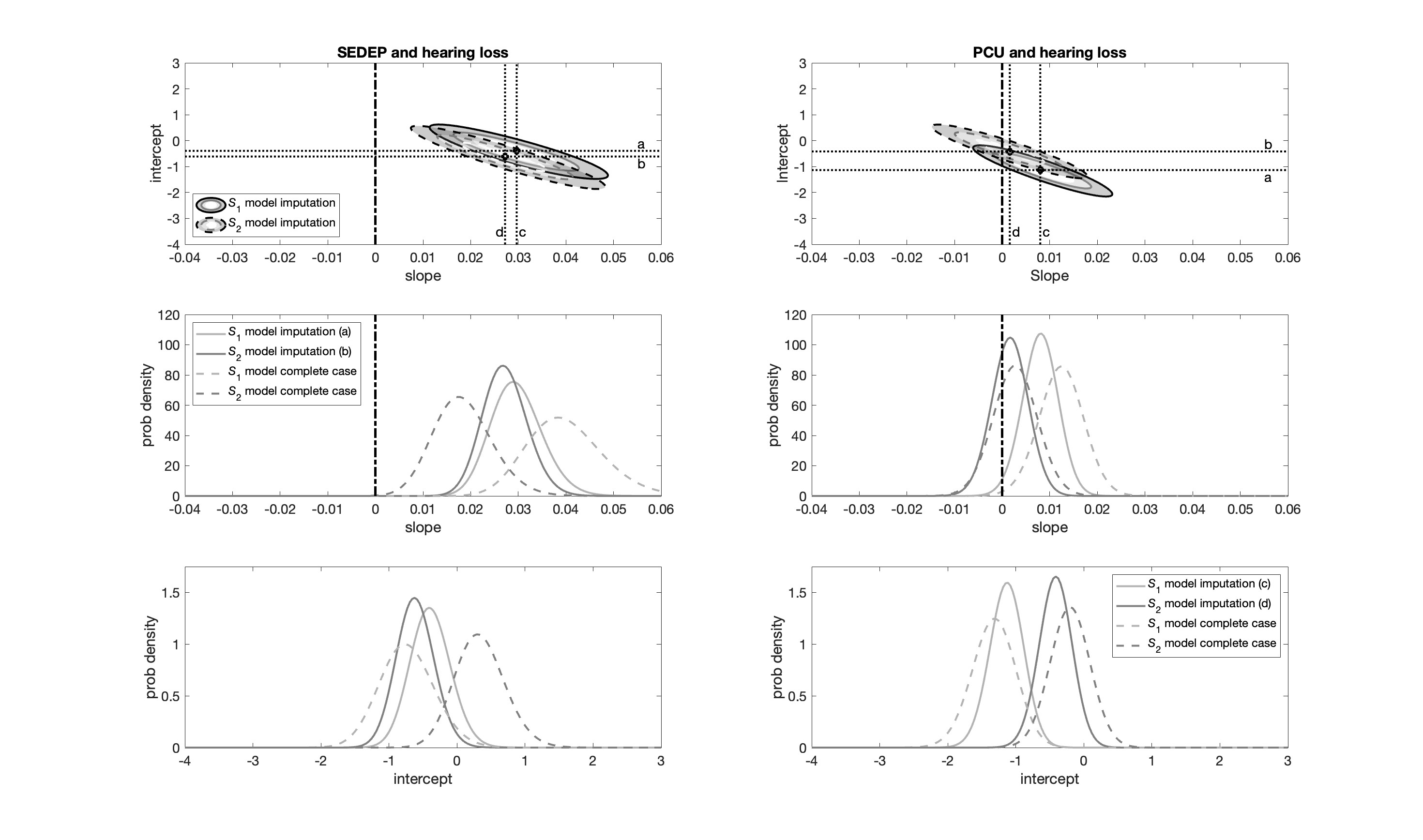

Supplement: Supplementary file 5 [file Image_2.jpg]

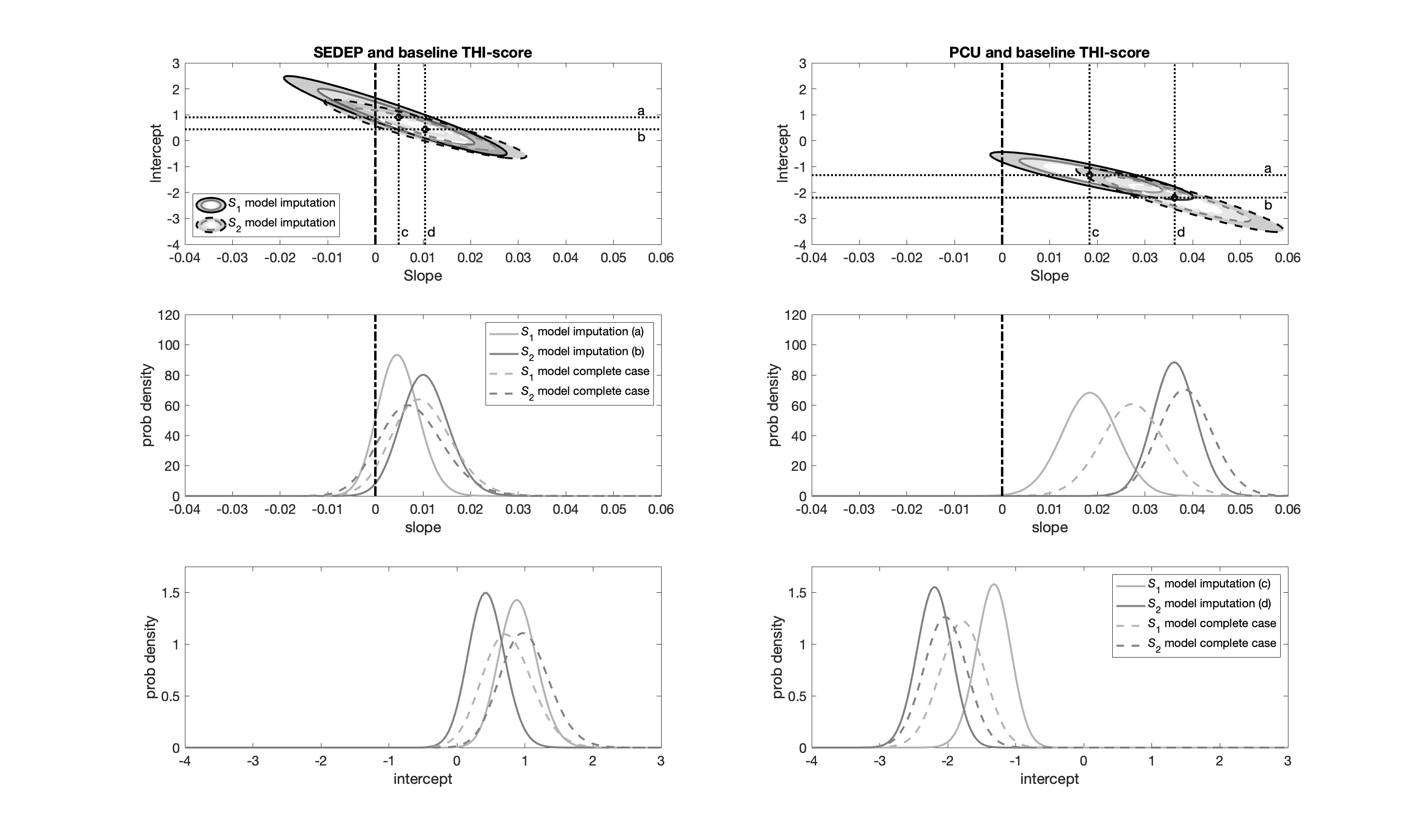

Supplement: Supplementary file 6 [file Image_3.jpg]
